# Mitochondrial Transcription Terminator Family Members mTTF and mTerf5 Have Opposing Roles in Coordination of mtDNA Synthesis

**DOI:** 10.1371/journal.pgen.1003800

**Published:** 2013-09-19

**Authors:** Priit Jõers, Samantha C. Lewis, Atsushi Fukuoh, Mikael Parhiala, Simo Ellilä, Ian J. Holt, Howard T. Jacobs

**Affiliations:** 1 Institute of Biomedical Technology and Tampere University Hospital, Tampere, Finland; 2 Estonian Biocentre, Tartu, Estonia; 3 Department of Biology, University of California, Riverside, California, United States of America; 4 Department of Clinical Chemistry and Laboratory Medicine, Kyushu University Graduate School of Medical Sciences, Fukuoka, Japan; 5 Department of Medical Laboratory Science, Junshin Gakuen University, Fukuoka, Japan; 6 MRC National Institute of Medical Research, London, United Kingdom; 7 Molecular Neurology Research Program, University of Helsinki, Helsinki, Finland; University of Cologne, Germany

## Abstract

All genomes require a system for avoidance or handling of collisions between the machineries of DNA replication and transcription. We have investigated the roles in this process of the mTERF (mitochondrial transcription termination factor) family members mTTF and mTerf5 in *Drosophila melanogaster*. The two mTTF binding sites in *Drosophila* mtDNA, which also bind mTerf5, were found to coincide with major sites of replication pausing. RNAi-mediated knockdown of either factor resulted in mtDNA depletion and developmental arrest. mTTF knockdown decreased site-specific replication pausing, but led to an increase in replication stalling and fork regression in broad zones around each mTTF binding site. Lagging-strand DNA synthesis was impaired, with extended RNA/DNA hybrid segments seen in replication intermediates. This was accompanied by the accumulation of recombination intermediates and nicked/broken mtDNA species. Conversely, mTerf5 knockdown led to enhanced replication pausing at mTTF binding sites, a decrease in fragile replication intermediates containing single-stranded segments, and the disappearance of species containing segments of RNA/DNA hybrid. These findings indicate an essential and previously undescribed role for proteins of the mTERF family in the integration of transcription and DNA replication, preventing unregulated collisions and facilitating productive interactions between the two machineries that are inferred to be essential for completion of lagging-strand DNA synthesis.

## Author Summary

All genomes require a system for preventing collisions between the machineries of DNA replication and transcription. We have investigated the roles in this process of two proteins of the mTERF (mitochondrial transcription termination factor) family in *Drosophila*. These factors, mTTF and mTerf5, share common binding sites in the mitochondrial genome, which we found to coincide with sites of replication pausing. Knockdown of either factor by RNA interference resulted in mtDNA depletion and developmental arrest. mTTF knockdown decreased site-specific replication pausing, but led to an increase in random stalling and regression of replication forks, with impaired synthesis of the lagging strand. This we attribute to random collisions with the transcriptional machinery. Conversely, mTerf5 knockdown led to enhanced replication pausing at mTTF binding sites. These findings indicate an essential and previously undescribed role for proteins of the mTERF family in the integration of transcription and DNA replication, preventing unregulated collisions and facilitating productive interactions between the two machineries that are inferred to be essential for completion of lagging-strand DNA synthesis.

## Introduction

The mitochondrial genome and its expression are essential to maintain oxidative phosphorylation (OXPHOS), a central metabolic process in higher eukaryotes. OXPHOS failure during development leads to developmental arrest in a diverse range of metazoans, including both insects [1], [2] and vertebrates. In the mouse, for instance, ablation of genes required for mitochondrial DNA (mtDNA) maintenance results in lethality at embryonic day 8–9 [3]–[5]. OXPHOS dysfunction also underlies many pathological states in humans [6]. Elucidation of the mechanisms of faithful mitochondrial genome maintenance and expression is therefore of both developmental and medical relevance [6].

In metazoans, mtDNA replication has been most extensively studied in mammals, where several competing models have been proposed. The strand-displacement model [7], originally based on imaging and end-mapping studies (see also [8]–[10]), contrasts with the evidence from two-dimensional neutral agarose gel electrophoresis (2DNAGE) analyses [11]–[16], supporting various types of strand-coupled replication. In the strand-displacement model, leading-strand synthesis initiates in the major non-coding region (NCR), at a site designated as the origin of heavy-strand synthesis (O_H_) [12], [13]. It then proceeds two-thirds of the way around the circle until reaching the site designated as the origin of light-strand synthesis (O_L_). Synthesis of the two strands on this model is asynchronous, but continuous on both strands, i.e. without a need for Okazaki fragments.

2DNAGE was developed almost three decades ago, to separate and characterize branched from linear DNA [17]. It has been widely used to analyze replication intermediates, starting in 1987 with the yeast 2 µ plasmid [18], and subsequently in hundreds of other publications. The method is considered definitive for inferring replication mode and origins, termination sites, fork barriers and molecular recombination (for review see [19]–[23]). 2DNAGE has indicated the existence of two classes of strand-coupled replication intermediate in mammalian mtDNA, which have been suggested to reflect alternate modes of replication that may operate in parallel. In the unidirectional RITOLS mode (RNA Incorporation Throughout the Lagging Strand), a provisional lagging-strand, consisting of RNA segments derived from processed transcripts, is established as the replication fork proceeds [14]. This RNA is then replaced by DNA in a subsequent maturation step, in which lagging-strand DNA synthesis is initiated at one or more preferred sites, including O_L_. RITOLS shares many features with the strand-displacement model, the only major difference being that the latter postulates that the parental strand displaced during heavy-strand replication remains single-stranded until the light-strand initiates. The second type of replication intermediate detected by 2DNAGE is composed fully of dsDNA, whose structure implies bidirectional initiation of replication across a wider origin zone, stretching beyond the NCR. However, termination at O_H_ means that this mode of replication is also effectively unidirectional [11], [16].

**Figure 1:**
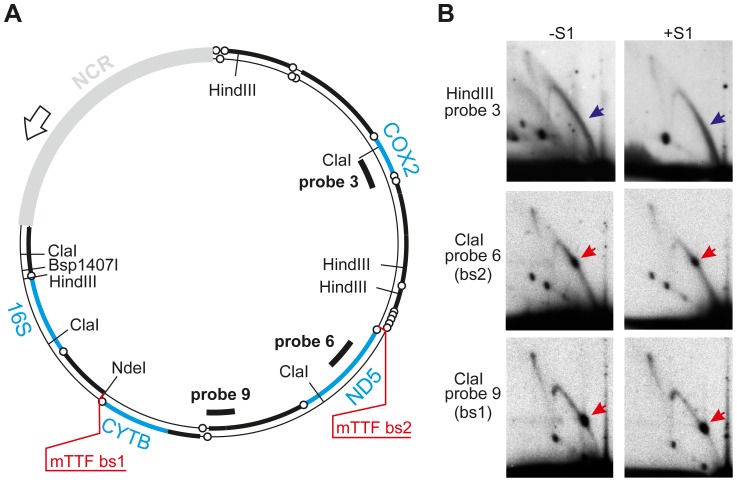
Replication pauses at mTTF binding sites. A. Schematic map of *D. melanogaster* mtDNA with positions of probes, mTTF binding sites (bs1, bs2), gene clusters (bold), tRNA genes (open circles), non-coding region (NCR, grey) origin and direction of replication (open arrow) and restriction endonuclease sites for Hind III, Cla I, Nde I and Bsp 1407I. Positions of genes for which expression was analyzed are shown in blue. B. 2DNAGE of ClaI- or HindIII-digested mtDNA. Red arrows indicate discrete spots on standard Y-arcs, representing major pause sites (replication fork barriers), analogous with those documented previously in other systems [59], [88], [89]: (see also relevant reviews cited in text, explaining the species seen by 2DNAGE). Blue arrows denote broader replication slow-zone in the HindIII fragment detected by probe 3. For more accurate mapping of pause sites by multiple digests, see Fig. S1.

Mitochondrial DNA replication in *Drosophila melanogaster*, based both on early TEM [24], [25] and more recent 2DNAGE analyses [26], also involves two replication modes. The majority of replication intermediates are composed entirely of dsDNA, with no evidence of extensive RNA incorporation. Their structure implies unidirectional strand-coupled DNA synthesis, commencing in the NCR, with an initiation site as inferred previously by end-mapping [27]. A minority of replicating molecules retain a region of single-strandedness encompassing the rRNA gene locus just downstream of the origin, indicative of delayed lagging-strand completion in this limited part of the genome. Also of note was the inference of specific replication pause regions through which the replication fork travels more slowly, based on the pronounced accumulation of replication intermediates containing fork structures therein. The two major pause regions of the mitochondrial genome [26] correspond approximately with zones of convergence of oppositely transcribed blocs of genes (Fig. 1A).

The coding region of metazoan mtDNAs shows a highly compact organization, with little or no non-coding sequences between genes. Typically, genes are encoded on both strands, a type of organization that unavoidably risks encounters between the transcription and replication machineries, which compete for the same template. As in other genetic systems, these processes should be subject to regulation, in order to minimize and resolve potential conflicts, including both co-directional and anti-directional collisions between the two molecular machineries. Defects in this collision regulation have been shown to cause abortive DNA synthesis, mutagenesis and genomic instability in a wide range of organisms [28]–[33]. In *E. coli*, for example, transcription termination is essential for the maintenance of genome integrity [34], by minimizing the generation of double-strand breaks arising from replication-fork collapse. A recent report has documented the importance of a machinery to regulate replication pausing caused by collisions with transcription complexes [35].

The mitochondrial transcription termination factor (mTERF) family comprises a set of mitochondrial DNA-binding proteins with diverse, documented roles in mitochondrial gene expression [36], [37]. The key structural feature of these proteins is the presence of multiple TERF motifs (I–IX), which have been shown, at least in the case of human MTERF1 and MTERF3, to form left-handed helical repeats that create a superhelical DNA-binding domain [38], [39]. mTERF family members have been implicated in the regulation of transcriptional initiation [4], [40], [41] as well as attenuation [40], [42], [43], and have also been shown to participate in mitoribosome assembly and translation [44]–[47]. In the mouse, *Mterf3* and *Mterf4* are essential genes [4], [45], while *Mterf2* is not [41]. Human MTERF1 terminates transcription bidirectionally *in vitro* at its major binding site downstream of the rRNA genes [48]–[50], but manipulation of its activity in cultured cells or knockout mice has rather modest effects on transcript levels [43], [51], whose physiological significance, if any, is unknown.

Four proteins of this family have been identified in *Drosophila*, of which the best characterized is mTTF (CG18124). mTTF binds two sequence elements in *Drosophila* mtDNA [42], each located at the junctions of convergently transcribed blocs of genes (see Fig. 1A). Its binding facilitates transcriptional termination bidirectionally *in vitro* and is required for transcriptional attenuation *in vivo* [52], [53]. The amount and activity of mTTF therefore influences the steady-state levels of mitochondrial RNAs whose coding sequences lie between the mTTF binding sites and the putative promoters [52]. Knockdown of an insect-specific paralog of mTTF, mTerf5 (CG7175), was found to have opposite effects on transcript levels to knockdown of mTTF, despite the fact that mTerf5 binds to the same sites in mtDNA in an mTTF-dependent manner [54].

As DNA binding proteins with an established role in the regulation of transcription, mTERF family members are strong candidates for mediating conflicts between the replisome and transcription complexes. Moreover, MTERF proteins may have multiple roles in mtDNA metabolism, considering that alterations in the levels of MTERF1 or its homologs MTERF2 (MTERFD3) and MTERF3 (MTERFD1) were reported to modulate the levels of paused replication intermediates in cultured human cells [55], [56]. The sea urchin mTERF protein mtDBP has been demonstrated *in vitro* to have contrahelicase activity [57]. This feature, commonly seen in replication termination proteins, is shared also by the mammalian nuclear rDNA transcription terminator TTF-1, which has been suggested to regulate entry of the replication machinery into an actively transcribed region [58]. The possible correspondence of the mTTF binding sites in *D. melanogaster* mtDNA with the regions of replication pausing identified in our earlier study suggests that mTERF family proteins could be considered as candidates for a similar role.

To test the possible involvement of mTTF and mTerf5 in mtDNA replication, we investigated their effects on mtDNA metabolism after manipulation of their expression by RNAi, both in cultured cells and *in vivo*. Here we show that both factors are required for normal mtDNA topology and maintenance. Lack of either (or both) resulted in developmental arrest at L3 larval stage. mTTF knockdown led to the accumulation of nicks, dsDNA breaks and recombination junctions. 2DNAGE demonstrated stalled and reversed replication forks over broad zones surrounding the mTTF binding sites, and an accumulation of aberrant replication intermediates with extended segments of RNA/DNA hybrid, indicating a failure to complete lagging-strand DNA synthesis. Knockdown of mTerf5 had an opposite effect on mtDNA replication intermediates, bringing about an increase in replication pause strength when compared to wild-type, a decrease in fragile replication intermediates containing single-stranded segments, and the disappearance of species with even the short segments of RNA/DNA hybrid that we were able to detect in wild-type cells.

Because of their opposing but essential roles in mtDNA expression and synthesis, we propose that the balance of these two mTERF family members facilitates the orderly and productive passage of oppositely moving replication and transcription complexes, preventing collisions that would otherwise result in abortive replication and loss of genome integrity.

## Results

### Replication pauses at mTTF binding sites

Replication pauses are revealed as discrete spots on arcs of replication intermediates resolved by 2DNAGE [17], [59]. The two major replication pause regions of *D. melanogaster* mtDNA were previously localized to approximately 1/3 and 2/3 of genome length from the replication origin, located in the NCR [26]. In order to map these pause sites more precisely, we conducted 2DNAGE on overlapping short restriction fragments, in a size range considered optimal for resolution on the standard two-dimensional gel system, i.e. 3–5 kb [60]. These analyses revealed the pause sites as the expected discrete spots (Figs. 1, S1, red arrows), lying on standard Y-arcs which are characteristic of non-origin fragments through which a replication fork passes unidirectionally (see [19]–[23], [60] for full explanations of the signals seen on 2DNAGE). Within the ∼50 bp resolution of the method, and based on multiple digests (Fig. S1), each pause maps precisely to one of the two binding sites for mTTF in the genome, namely at the ND1/tRNA^Ser(UCN)^ gene boundary (here designated bs1) and the tRNA^Phe^/tRNA^Glu^ gene boundary (bs2; see Fig. S1C for explanation of mapping). The HindIII fragment beyond bs2, encompassing the remainder of the coding region, did not reveal any discrete pause signals. However, an enhanced signal relative to that seen in the adjacent ClaI fragments was evident at the start of the Y arc in this fragment (blue arrow), suggestive of a more diffuse replication slow-zone at the origin-proximal end of this fragment, consistent with previous data [26]. Treatment with the single strand-specific nuclease S1 had no effect on the migration of replication intermediates in any of the fragments tested, consistent with the previous inference that DNA replication in these regions is fully strand coupled [26].

### mTTF knockdown in S2 cells causes mtDNA depletion and altered topology

**Figure 2:**
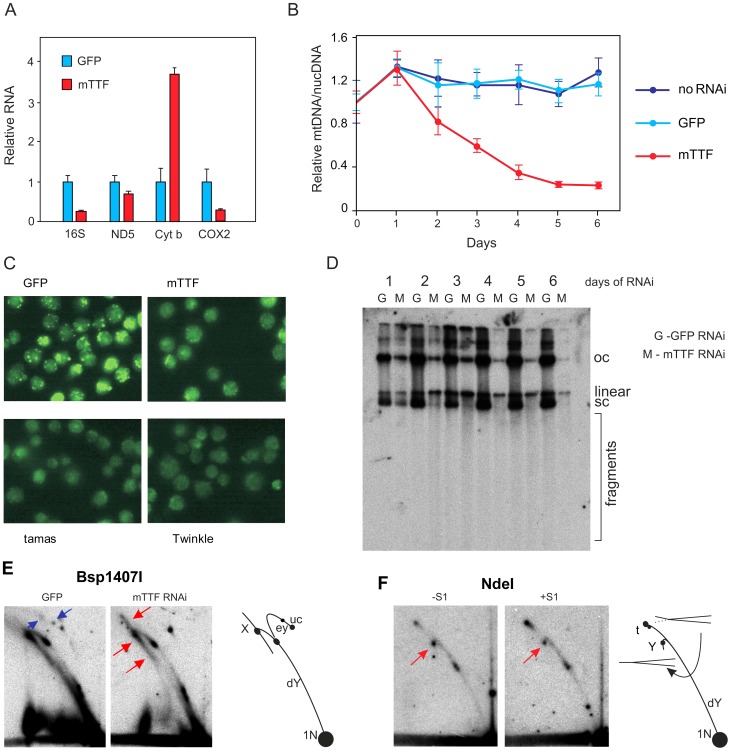
Effects of mTTF knockdown in S2 cells. A. Mitochondrial transcript levels after 3-RT-PCR. Means ± SD, normalized to values for the cells treated with the inert control dsRNA against GFP. B. mtDNA copy number changes measured by qPCR, following treatment with dsRNA against GFP, mTTF and mock-transfected cells from three independent replicates (means ± SD, normalized to value for cells prior to plating). C. Images of PicoGreen-stained cells after 3 d of dsRNA against GFP, mTTF, tamas and CG5924 (*D. melanogaster* homologue of mitochondrial helicase Twinkle). D. Agarose gel-blot of uncut mtDNA, hybridized with probe 3, showing three major forms (oc – open circles, linear – genome-length linears, sc – supercoiled circles). Note the relative disappearance of sc forms, increase in linear forms and subgenomic fragments, in cells treated with dsRNA against mTTF (M), compared with cells treated with inert control dsRNA against GFP (G). Note also that this experiment is not quantitative, since it uses DNA extracted from isolated mitochondria, for which a nuclear DNA loading control is meaningless. E, F. 2DNAGE of mtDNA cut by restriction enzymes with single digestion sites in the genome. E. Bsp 1407I: note the disappearance of the faint but characteristic, partially single-stranded eyebrow arc (blue arrows: for detailed explanation, see [26]) and the increase in recombination intermediates or X-forms (red arrows, [17]), after 3 d of mTTF dsRNA treatment. Drawing (right) shows and interprets the major arcs seen on the gel, based on published 2DNAGE analyses (see references cited in text). uc – uncut circles, ey – eyebrow arc, X – recombination arc, dY – double Y arc, 1N – unit length fragment (genome-length linear). F. Nde I digest, followed by treatment with S1 nuclease, or not, as indicated. Note the accumulation of replication intermediates broken at the origin/rRNA locus (compare with Fig. 6 of [26]: see Fig. S3). Drawing (right) shows the conversion of replication intermediates from X-like termination structures (t) to Y structures (Y) by strand breakage within the region depicted by the dashed line.

The coincidence of replication pauses with the previously mapped binding sites for mTTF suggests a role for this protein in mtDNA maintenance. To investigate this we used dsRNA-based RNA interference to knock down mTTF in S2 cells. A ∼70% decrease in mTTF mRNA levels (Fig. S1A) resulted in altered mitochondrial transcript levels consistent with the previous report by Roberti et al. [52]. Depending on their location within the transcription map, transcripts were either upregulated (e.g. cytochrome b mRNA), downregulated (e.g. 16S rRNA and COX2 mRNA), or little altered (e.g. ND5 mRNA) (Fig. 2A). Furthermore, in untreated cells, transcript levels of a given strand were observed to decrease markedly as the mTTF binding sites are successively traversed within the transcription unit (Fig. S1B), consistent with the proposed role of mTTF as a transcriptional attenuator (although this may also be influenced by differential RNA stability).

Next we analyzed mtDNA levels in cells knocked down for mTTF, using three different methods: qRT-PCR (Fig. 2B), PicoGreen staining of mtDNA nucleoids (Fig. 2C) and Southern blotting of both digested (Fig. S1C, D) and undigested mtDNA (Fig. 2D). qRT-PCR indicated that mtDNA levels fell to approximately 20% of control levels following 4–5 days of mTTF knockdown (Fig. 2B), whereas mtDNA levels were unchanged in untreated cells or cells treated with an inert dsRNA against GFP. The intensity of PicoGreen staining after 5 d of mTTF knockdown (Fig. 2C) was also greatly diminished compared with control cells treated with the dsRNA against GFP and similar to the effect of dsRNA treatment targeted against genes with well-established roles in mtDNA synthesis, such as *tamas* (encoding the catalytic subunit of DNA polymerase gamma) or CG5924 (the *Drosophila* homologue of the mammalian mitochondrial helicase Twinkle). The relative amount of intact mtDNA detected by Southern blot was also diminished by mTTF knockdown (Fig. 2D), with a progressive disappearance of the supercoiled form relative to genome-length linear molecules (Fig. 2D). The total amount of mtDNA detectable by Southern hybridization after digestion with a restriction enzyme was also diminished (Fig. S2C, S2D). Analysis of full-length mtDNA by 2DNAGE revealed a relative increase both of recombination structures and broken replication replication intermediates (Fig. 2E: see [26] for a full explanation of the arcs revelaed by 2DNAGE of *Drosophila* mtDNA digested with restriction enzymes curring once in the genome). Recombination structures linking two whole copies of the genome following such linearization are most easily revealed in the Bsp1407I digest, where the characteristic X-arc that they form (see [19]–[23], [60]) is well resolved from termination and dY structures. Their accumulation was most prominent after 3 d of knockdown of mTTF (Fig. 2E, red arrows). Broken replication intermediates, arising from scission of one branch at or near the origin, migrate on or close to a standard Y-arc, instead of a bubble, double-Y or eyebrow arc (see Figs. 2E, 2F, S3). They are normally found only at a low-level in control cells, but are generated in material from control cells by treatment with S1 nuclease, which cuts the region that remains single-stranded in some replicating molecules, extending from the replication origin across the rRNA gene locus (see Fig. 6 of [26], panels from which are reproduced here in Fig. S3 for comparison). After mTTF knockdown, these broken intermediates were much more abundant, and further treatment with S1 nuclease had no effect (Fig. 2F, red arrows). The characterstic eyebrow arc seen in the Bsp1407I digest, resulting from non-digestion in the partially single-stranded region, was already absent, consistent with systematic strand-breakage in this region following mTTF knockdown (Fig. 2E, blue arrows).

Roberti et al. [52] earlier found no significant effect on mtDNA levels from 3 days of mTTF knockdown, but using a different dsRNA. To clarify this inconsistency and exclude possible off-target effects, we repeated the experiment using either the same dsRNA as Roberti et al. [52] or our own custom-designed dsRNA. mtDNA levels were decreased by ∼80% at day 5 in both cases, although the dsRNA used by Roberti et al. [52] produced its effects more slowly, with only a 15% drop in mtDNA levels at day 3 (Fig. S2E).

### mTTF knockdown in developing flies causes mtDNA depletion, broken replication intermediates and larval arrest

**Figure 3:**
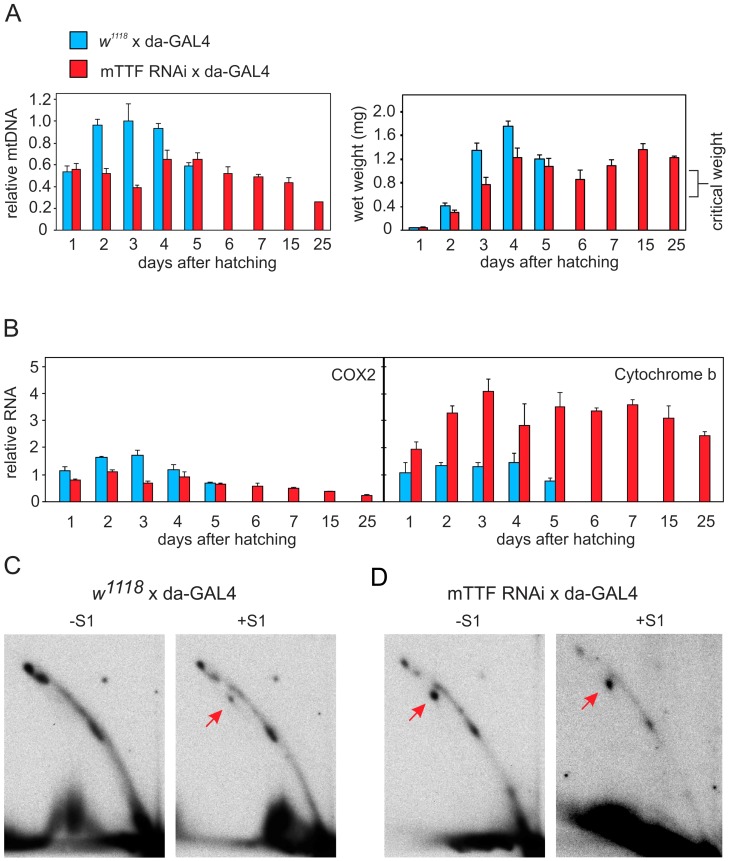
Effects of mTTF knockdown in flies. A. Changes in mtDNA copy number, measured by qPCR and normalized to value from control larvae at day 3, and in wet weight, of larvae from strains knocked down for mTTF (red bars, *w^1118^* ; *UAS-mTTF-RNAi*/+ ; *da-GAL4*/+) and controls (blue bars, *w^1118^* ; +/+ ; *da-GAL4*/+) from three independent replicates. Means ± SD. Critical weight is the threshold for progression to metamorphosis. B. Expression of COX2 and cyt b genes in larvae from mTTF knockdown (red bars) and control strains (blue bars), measured by Q-RT-PCR from three independent biological replicates. Means ± SD. C, D. 2DNAGE of NdeI-digested mtDNA with or without S1 treatment, in (C) control and (D) mTTF knockdown larvae, as indicated. Note the appearance of broken replication intermediates (red arrows) in mTTF knockdown larvae which are only visible in material from control larvae after S1 treatment (compare with Fig. 4 of [26]; see also Figure 2F).

To investigate the effects of mTTF knockdown on mtDNA maintenance in the whole organism, we expressed a (hairpin) dsRNA transgene targeted on mTTF, using the ubiquitous and constitutive *daughterless-*GAL4 (da-GAL4) driver. We confirmed that the parental RNAi line (itself homozygous viable) produced normal numbers of progeny with a wild-type phenotype when mated to flies not expressing da-GAL4. We also confirmed that RNA interference *in vivo* produced ∼90% knockdown of mTTF at the mRNA level at larval stage (Fig. S4A), which was seen also at the protein level (Fig. S4B). mTTF knockdown larvae gained weight more slowly than wild-type larvae of the same genetic background (Fig. 3A). More than 90% of individuals failed to develop beyond the L3 larval stage although larval weight exceeded the critical range for developmental progression (Fig. 3A, [61]]. None of the few aberrant pupae progressed to the late pupal stages. The persistent larval stage lasted approximately 30 days, during which the larvae became progressively inactive and then died.

Mitochondrial RNA levels were altered in a similar manner as in mTTF knockdown cells, e.g. COX2 mRNA was decreased, whereas cytochrome b mRNA was elevated (Fig. 3B). Mitochondrial DNA copy number failed to increase as typically occurs during wild-type development, remaining at 40% of the wild-type level 3 days after hatching (Fig. 3A). During the persistent larval stage, mtDNA copy number steadily declined to approximately 25% of the maximum level observed in wild type L3 larvae, 25 days after hatching.

A similar accumulation of broken replication intermediates was observed in mTTF knockdown larvae as in S2 cells, e.g. as revealed by NdeI digestion (Fig. 3D, red arrows; compare with Figs. 2F, S3). The control strain (*w^1118^* ; *da-GAL4*/+) displayed an identical pattern of replication intermediates to that described previously for the Oregon-R wild-type strain (Fig. 4 of [26]], ruling out any confounding effect of genetic background.

### mTTF knockdown causes replication stalling in a broad zone, with failure to complete lagging-strand synthesis

**Figure 4:**
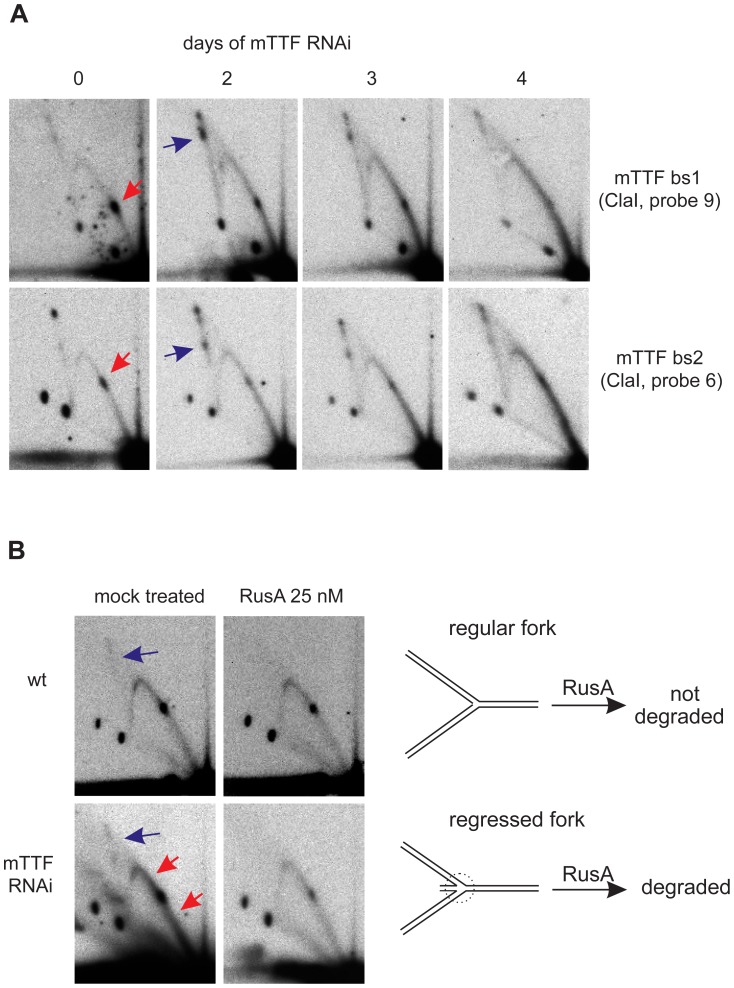
Aberrant replication-fork stalling resulting from mTTF RNAi. A. 2DNAGE of ClaI-digested mtDNA, time-course during mTTF dsRNA treatment, showing effects on replication intermediates in fragments containing the mTTF binding sites, i.e. substitution of defined pauses (red arrows) with wider regions of stalling across both mTTF binding sites. B. 2DNAGE analysis of replication intermediates of fragment containing mTTF bs2 before and after RusA treatment. Note that RusA promotes increased removal of signal from the Y-arc (red arrows) in material from cells subjected to mTTF dsRNA treatment, as well as the disappearance of X-forms in both cases (blue arrows). Drawing illustrates how regressed replication forks become sensitive to cruciform-cutting enzymes. See also Figs. S6 for fuller explanation of RusA action and S7 for side-by-side comparisons, documenting increased sensitivity to RusA after mTTF knockdown.

The observed drop in mtDNA copy number and topological changes following mTTF knockdown prompted us to characterize mtDNA replication intermediates in more detail, in cells and larvae knocked down for mTTF. In each of the two ClaI fragments that contained the mTTF binding sites, the discrete spots corresponding to replication pauses were observed to fade out and spread over a wider region of the Y-arc, during 4 days of mTTF dsRNA treatment of S2 cells (Fig. 4A, red arrows). After 4 days of treatment, the proportion of this novel material migrating along the Y-arc, relative to the unit-length fragment, was significantly increased compared to day zero for both bs1 and bs2. Concomitantly we observed a transient increase in cruciform DNA species, particularly a subclass of Holliday junction-like molecules (Fig. 4A, blue arrows). This is consistent with the increase in recombinational forms linking two full copies of the genome seen after 2–3 days of RNAi following digestion with restriction enzymes that cut once in the genome (Fig. 2E, red arrrows). Spreading of the pauses (Fig. S5, red arrows), with accumulation of recombination junctions (Fig. S5, blue arrows) was seen in mTTF knockdown larvae, although to a lesser extent than in mTTF knockdown cells.

Stalled replication forks have a tendency to regress along the template and, under some conditions, can adopt a “chickenfoot” structure around a Holliday-like junction [62], [63]; (see Figs. 4B and S6). If such fork reversal is relatively limited, the species formed would still migrate close to a classical Y-arc. However, they should become sensitive to nucleases targeting Holliday junctions [64]. To test this, we treated mtDNA with the bacterial cruciform-cutting enzyme RusA. This removed substantially more material from the region of the Y-arc in cells knocked down for mTTF compared to control cells (Fig. 4B, red arrows; see also Fig. S7 for side-by-side comparisons of gels at equivalent exposures). This supports the idea that mTTF knockdown resulted in the accumulation of regressed replication forks containing Holliday-like junctions, which may be considered a signature of replication stalling. Note the decrease of the recombination structures (blue arrows in Fig. 4B) migrating on the X-arc, following RusA treatment, thus confirming the functionality of the enzyme in this experiment.

**Figure 5:**
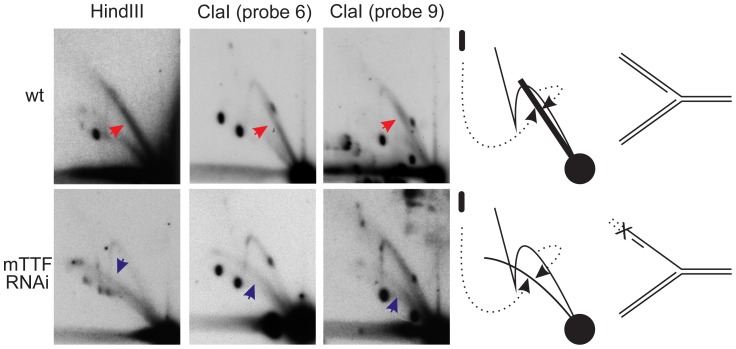
mTTF knockdown lengthens RNA/DNA hybrid tracts in replication intermediates. 2DNAGE analysis of RNase H-treated replication intermediates from untreated and mTTF knockdown cells (after 3 d of treatment). Note the appearance in material from control cells of an arc migrating close to the standard Y arc (red arrows, bold line in interpretative diagram, right), corresponding with structures containing limited segments of single-strandedness, as shown, arising from RNA removal from short regions of RNA/DNA hybrid. The novel Y-like arc generated in mTTF knockdown cells (blue arrows) follows a much shallower trajectory, indicative of more single-strandedness, ascribed to the presence of more extensive RNA/DNA hybrid segments prior to RNase H digestion. Drawing shows the structure and provenance of the novel arcs (from material previously unable to enter the gel).

Our findings are consistent with the idea that bound mTTF provides a natural barrier to fork progression, avoiding unregulated replication stalling that might arise, for example, from collisions of the replication and transcription machineries. Since mTTF is already known to promote transcriptional termination, we reconsidered the issue of the role of RNA in *Drosophila* mtDNA replication. Previous 2DNAGE analyses indicated that mtDNA replication intermediates in *D. melanogaster* were fully double-stranded [16], except for the rRNA locus, which exhibited single-strandedness in a minority of molecules. Restriction sites across the remainder of the genome were completely digestible, indicating that extensive regions of RNA/DNA hybirid, such as seen in RITOLS replication intermediates in vertebrates [12], [13], [65], were absent, although the presence of limited patches of RNA/DNA hybrid could not be excluded. We investigated the issue further by treating mtDNA, after restriction digestion, with RNase H, which digests regions of RNA hybridized to DNA, thus modifying the migration pattern of RITOLS-type replication intermediates. This analysis revealed a prominent, novel arc, migrating just below the Y-arc (Fig. 5, red arrows), whose trajectory is consistent with the presence of one or more short segments of ssDNA, arising from the enzymatic removal of RNA from some replicating molecules. Other species detectable by 2DNAGE were essentially unaffected by RNase H treatment, indicating that the novel arc arose from material previously not resolved on this gel system, which is consistent with the clear increase in signal seen after RNase H treatment (Fig. S8).

The nature and trajectory of the novel arc released by RNase H treatment differed markedly after knockdown of mTTF. The forms migrating just below the standard Y-arc (Fig. 5, red arrows), were replaced by a much shallower sub-Y arc, extending beyond the limit of the fragment analysed (Fig. 5, blue arrows). Its trajectory is consistent with much more extensive ssDNA regions (i.e. much longer segments of RNA/DNA hybrid prior to RNase H treatment) than in the replication intermediates that formed the sub-Y arc generated by RNase H treatment in untreated cells.

### mTerf5 is also required for mtDNA maintenance

**Figure 6:**
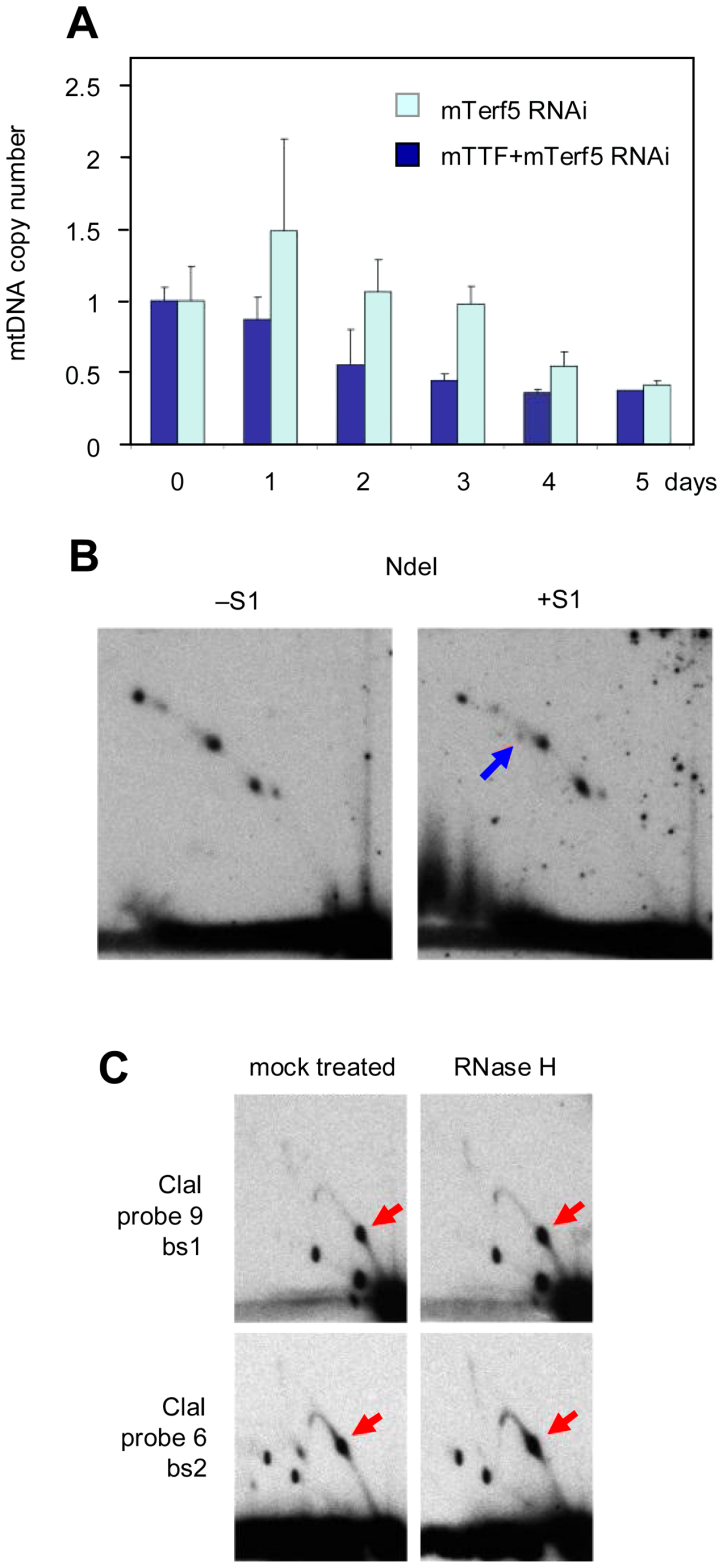
Effects of mTerf5 knockdown in S2 cells. A. mtDNA copy number changes, measured by qPCR, following treatment with dsRNAs against mTerf5 alone, or against both mTTF and mTerf5. Means ± SD from three independent experiments, normalized against values prior to plating (day 0). B. 2DNAGE of NdeI-digested mtDNA after 3 d of treatment with dsRNA against mTerf5: compare with Figure 2F and Fig. 6 of [26] (see Fig. S3B for side-by-side comparisons). Blue arrow denotes broken replication intermediates, now barely visible even after treatment with S1 nuclease. C. 2DNAGE of ClaI-digested mtDNA with or without subsequent digestion with RNaseH, after 3 d of treatment with mTerf5 dsRNA: compare with Figure 4A. Red arrows denote enhanced major pauses corresponding with bs1 and bs2: see Fig. S10 for side-by-side comparisons documenting the increase in signal.

To test whether the mTTF partner protein mTerf5 antagonizes the effects of mTTF on replication as well as on transcription, we investigated the effect of mTerf5 knockdown on mtDNA copy number in S2 cells (Fig. 6A). We observed a substantial depletion of mtDNA to a similar extent (∼70%), and with similar kinetics, as mTTF knockdown, although there was no cross-reaction between the two dsRNAs used (Fig. S9). Simultaneous knockdown of both factors in S2 cells produced a small initial increase in mtDNA copy number, followed by a gradual decline to the same low level as produced by knockdown of either factor alone, after 5 days of treatment. In the developing fly, mTerf5 knockdown using each of three independently isolated RNAi lines driven by da-GAL4, produced the same phenotype as mTTF knockdown, i.e. a persistent larval stage with failure of pupariation. The congruent phenotype effectively excludes off-target effects as an explanation. Simultaneous knockdown of both factors in the developing fly also yielded this phenotype.

Despite the fact that mTerf5 knockdown produced similar effects on mtDNA copy number and development as mTTF knockdown, 2DNAGE analysis of mtDNA from mTerf5 knockdown cells revealed different effects on the pattern of replication intermediates. We observed enhanced replication pausing at both mTTF binding sites (Fig. 6C: for comparison based on gels of equivalent exposure, see Fig. S10). The broken intermediates that accumulated when mTTF was knocked down were absent (compare Fig. 6B with Fig. 2F, shown alongside in Fig. S3B), and treatment with S1 nuclease failed to release such intermediates in comparable amounts as in control cells (Fig. 6B, Fig. S3B). Treatment with RNase H had no discernible effect (compare Fig. 6C with the corresponding digests of Fig. 5). mTerf5 knockdown thus had opposite effects on replication intermediates as mTTF knockdown, appearing to enhance replication pausing at specific sites and shifting the balance of mtDNA replication intermedaites towards those composed fully of dsDNA, as opposed to those with patches of RNA/DNA hybrid or single-strandedness.

## Discussion

mTTF and mTerf5 were previously shown, using RNAi, to have reciprocal effects on transcription. Here we investigated their roles in mtDNA maintenance, using a similar strategy. Both factors were essential for mtDNA copy number maintenance, but had opposing effects on mtDNA replication. These findings allow us to propose a model whereby these factors co-operate to facilitate the productive interaction between oppositely moving replication and transcription complexes on the same template, thus contributing to the maintenance of genomic fidelity.

Contrary to a previous report [52], our data demonstrate that mTTF is required *in vivo* to maintain mtDNA levels. The different findings are attributable to the kinetics of action of the dsRNAs used in the two studies. The effects on transcription were broadly similar [36]: the minor differences are most likely due to early changes in mtDNA levels compounding those on RNA. The apparent drop in steady-state transcript levels as the mTTF sites are successively traversed reflects the organization of the mitochondrial genome, but makes no obvious sense for the equimolar supply of polypeptides belonging to any given OXPHOS complex. The transcription termination activity of mTTF might therefore serve primarily a different role, such as in DNA replication, with effects on mitochondrial transcripts being accommodated (post-) translationally.

The developmental arrest at larval L3 stage caused by deficiency of mTTF or mTerf5 is a phenotype shared by knockdown of many genes for mitochondrial functions, including those encoding mitochondrial transcription factor 2 (mtTFB2), single-strand binding protein (mtSSB) and CCDC56, required for the assembly of cytochrome c oxidase [66]–[68]. Whether it is a direct result of OXPHOS deficiency or of deranged developmental signaling remains to be determined.

Although we previously found no evidence for RNA-containing mtDNA replication intermediates in *Drosophila* [26], finer scale analysis indicated the presence of short patches of RNA scattered around the mitochondrial genome, based on the prominent sub-Y arcs seen on 2DNAGE after treatment with RNase H. Standard Y-arcs, which were already present before RNase H treatment, also remained after the treatment. There was a clear and reproducible increase in total signal in the resolving portion of 2D gels following treatment with RNase H. Logically, this material must have been released by the specific action of the nuclease, from high molecular-weight complexes or tangles previously unable to enter the gel, This is supported by similar observations on human heart mtDNA [69], much of which remained trapped in the well upon 2DNAGE, unless treated with suitable nucleases and/or topoisomerases to disrupt tangles visualized also by electron microscopy. We infer that mtDNA replication intermediates in *Drosophila* must consist of two classes, as in vertebrates. One class appears to be composed entirely of dsDNA, and is represented by the standard Y-arcs seen both before and after RNase H treatment. The second class, akin to the RITOLS intermediates seen in vertebrates [12], [13], contains tracts of RNA/DNA hybrid, except that here such tracts must be very short, so that RNase H generates a novel sub-Y arc which migrates close to the trajectory of the standard Y-arc. Short segments of RNA hybridized to replicating DNA may be covalently joined to longer transcripts, forming the complex tangles unable to enter gels unless released by RNase H treatment. The Y-like structure of the products, and the fact that they were created, not destroyed by RNase H, indicates that they are not simple intermediates of transcription, DNA repair or recombination.

These observations raise the issue of whether transcription and DNA replication can occur simultaneously on the same template and, if so, whether this association is obligatory. The existence of a population of mtDNA molecules only able to enter agarose gels after treatment with a ribonuclease strongly suggests that these are molecules engaged in active transcription. After RNA removal, they migrate along 2DNAGE arcs expected for an iterative set of replication intermediates, strongly supporting the idea that replication and transcription can take place on the same template. Those replication intermediates that can be resolved on 2D gels without ribonuclease treatment may represent a distinct subset of replicating molecules, in which transcription is prevented. Resolving these issues will require the development of novel methods for metabolic labeling and analysis of replication and transcription intermediates.

Knockdown of mTTF or mTerf5 produced specific and reciprocal effects on mtDNA synthesis. Lack of mTTF caused random stalling and fork regression, whilst decreasing those molecules specifically paused at the binding site itself. RusA treatment confirmed the presence of Holliday-like junctions, a signature of fork reversal associated with stalling due to replication impediments [63], and proposed as a necessary intermediate in replication repair [70]. The logical explanation for replication stalling in the present case is random collisions with the transcriptional machinery, as observed in other systems [29], [30], [71], such as the rDNA locus in yeast. The formation of Holliday-like chicken-foot structures at stalls of this type has not been reported previously, but our observation of an increase in X-form species containing recombination junctions after 2–3 days of mTTF knockdown suggests that stalling creates substrates for a recombinational repair and/or restart machinery. The observed mtDNA depletion and shift in topology indicates that such processes are unable to support the completion of replication sufficiently to maintain mtDNA copy number. The concomitant accumulation of broken replication intermediates, akin to those that can be created in material from unmanipulated cells by S1 nuclease treatment, indicates that the ssDNA region in the rRNA locus was systematically broken, suggesting that it was more pervasive or persistent than in control cells. Finally, a novel class of putative replication intermediates was observed to accumulate, inferred to contain more extensive RNA segments, based on the generation of shallower sub-Y arcs by RNase H. These replaced the forms with only short RNA segments, that were seen in control cells. Conversely, mTerf5 knockdown produced opposite effects, namely enhanced pausing at the mTTF binding sites, a decrease in replication intermediates broken at the rRNA locus and disappearance of the RNA-containing species. Thus, whereas mTTF is required for physiological pausing, mTerf5 allows paused replication to resume. Additional enzymatic treatments, as well as the use of in gel-digestion [72], heat denaturation prior to second dimension electrophoresis [73], [74], and electron microscopy, will be needed to reveal the detailed structural differences between replicating molecules paused naturally by mTTF/mTerf5, and those arising from unregulated or persistent stalling in their absence.

Some of the effects of mTTF knockdown on mtDNA replication could be indirect, e.g. resulting from altered transcript levels. However, a failure of replication due to primer insufficiency would lead to the progressive disappearance of shorter replication bubbles, rather than the accumulation of broken termination intermediates. Evidence of a role for preformed transcripts in RITOLS replication of mammalian mtDNA, via the bootlace mechanism [13], [14], suggests the possibility that mTTF deficiency might impair DNA replication by distorting the relative abundances of different processed transcripts that must be incorporated during fork progression. However, this would not explain the accumulation of random collision products. Thus, we favor a more direct role for mTTF in DNA replication.

**Figure 7:**
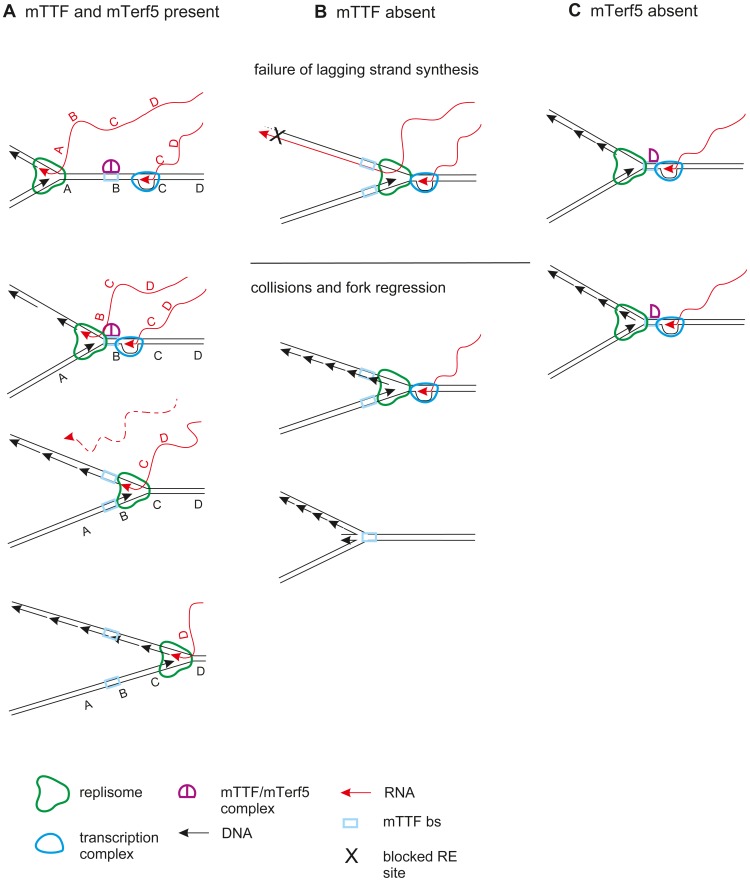
A tentative model for mTTF function. A. Normally, replication pauses at mTTF binding sites, where orderly passage of transcription and replication is mediated. mTTF binding sites may additionally be the places where an RNA bootlace is supplied [14], via termination of a nascent transcript produced by an arriving transcription complex. Note that, under this model, as the fork advances further, RNA/DNA hybrid is laid down behind the fork, whilst the replicative helicase unwinds the parental duplex ahead of the fork. The lagging-strand RNA can then be processed to generate primers for lagging-strand DNA synthesis, as the fork proceeds. Letters mark corresponding positions on RNA and DNA strands. B. In case of mTTF depletion by RNAi, uncontrolled collisions between the replication and transcription machineries take place outside of the mTTF binding site, leading to fork reversal and a failure of normal lagging-strand synthesis. C. Depletion of mTerf5 by RNAi is proposed to enhance the binding or inhibit the dissociation of mTTF, resulting in stronger pausing, a block to onward fork progression, and early completion of the lagging strand in the rRNA gene region.

The effects of mTTF and mTerf5 knockdown imply that RNA incorporation, replication-fork pausing and lagging-strand synthesis are related phenomena. RNA incorporated via the bootlace mechanism is one possible source of primers for the synthesis of lagging-strand DNA, although other mechanisms of lagging-strand initiation are consistent with RITOLS [9]. Our data suggest that proteins belonging to the mTERF family are crucial factors in execution and/or regulation of such a process, at least in *Drosophila*, as illustrated in Fig. 7. The proposed model postulates that the balance of mTTF and mTerf5 nurses the productive interaction of replication and transcription machineries moving in opposite directions, and that replication pausing is vital for ensuring the incorporation of RNA transcripts into replication intermediates at the replication fork (Fig. 7). Capture of a new bootlace, resulting from the arrival of a transcription complex that undergoes termination, is also proposed to be essential for the priming of lagging-strand DNA synthesis, not only at the immediate site of mTTF/mTerf5 binding, but also further downstream, as the replication fork progresses.

The prevention and/or regulation of collisions between the transcription and replication machineries is indispensable for all genetic systems [30], [75], to avoid knotting of the daughter molecules [76], the generation of recombinogenic ends [77] and other types of genomic instability [29]. Proteins that perform this function are well documented in other systems, for example in the rDNA of both fungi [78], [79] and mammalian cells [80], although these proteins (Fob1 in *S. cerevisiae*, Reb1 in *S. pombe* and TTF1 in mammals) are unrelated to the mTERF family and to each other. Thus, there is both a precedent and a rationale for mTTF and mTerf5 to integrate transcription and DNA replication.

However, the many cases of mitochondrial proteins having multiple functions, e.g. Ilv5, Aco1 and RNase P [81]–[83], means that it cannot be excluded that mTTF and mTerf5 regulate transcriptional and replication independently. The two proteins may also be considered as an example of an antagonistic pair that together control a specific process, a type of regulation widespread in biological systems. An intriguing parallel is provided by the related helicases Rrm3 and Pif1, which exert opposing effects on DNA synthesis at the replication fork barrier of *Saccharomyces cerevisiae* rDNA [84]. Unlike mTTF and mTerf5, Rrm3 and Pif1 do not bind DNA sequence-specifically, but can recognize and process unusual DNA structures in G+C-rich regions [85]. Although mTTF and mTerf5 are not themselves helicases, they may recruit an antagonistic helicase pair that act in a similar manner to Rrm3 and Pif1, or may confer alternate properties on a single helicase.

## Materials and Methods

### Flies, cell-lines and culture

S2 cells [86] were cultured in Schneider's Medium (Sigma-Aldrich) at 25°C. Cells were passaged every 3–4 d at a density of 0.5×10^6^ cells/ml. Standard *Drosophila* strains, plus the mTTF RNAi line 101656 and mTerf5 RNAi lines 2899, 2900 and 107227 from the Vienna Drosophila RNAi Center (VDRC), were cultured as described previously [87].

### dsRNA constructs and transfection

Gene-specific dsRNAs were synthesized from templates created from S2 cell cDNA by a nested PCR strategy, which introduced the T7 promoter sequence on both sides of each final amplicon (see Table S1 for primer sequences). S2 cells were transfected with 4 µg of each dsRNA added to 0.5 ml of culture medium, and grown for the times indicated in figures and legends. Where transfections were to be continued for >3 d, cells were passaged every 3 d and fresh dsRNA was added. For visualization of nucleoids, dsRNA against Tfam was added for the final 2 d, as described in SI, Nucleoids were detected by fluorescence microscopy, after staining with Quant-iT™ PicoGreen (Invitrogen).

### DNA and RNA extraction

Nucleic acids for mtDNA copy-number analysis, 2DNAGE and Q-RT-PCR were isolated from S2 cells, adult flies, larvae or purified mitochondria thereof using variants of standard methods. In general, 2DNAGE used total nucleic acids isolated from sucrose density gradient-purified mitochondria (see SI).

### Q-PCR

Q-RT-PCR to measure RNA levels was performed essentially as described earlier [52], using cDNA prepared by random priming or, where indicated, by gene- and strand-specific primers as detailed in Table S1. Assays always included three or more independent biological replicate samples, with normalization to the transcript of nuclear gene RpL32. Relative quantitation of mtDNA content was performed similarly, using total DNA as template, plus primers for mitochondrial 16S rDNA (Table S1), also with normalization to RpL32.

### 1D and 2D neutral agarose gel electrophoresis and Southern blot-hybridization

Standard one-dimensional electrophoresis used 0.6% agarose gels in TBE buffer. 2DNAGE and blot-hybridization were conducted essentially as described earlier [13], using slightly different conditions for resolving large and small DNA fragments (see SI). For details of restriction digests and treatment with DNA modifying enzymes see SI. Radioactive probes for specific fragments of *Drosophila* mtDNA were generated by PCR, with [α-^32^P]-dCTP (Perkin-Elmer, 3000 Ci/mmol) in the reaction mix (see Table S1 for primers).

For further details, see Text S1.

## Supporting Information

Figure S1 Location of replication pause sites coincides with mTTF binding sites. A. Map of *Drosophila* mitochondrial genome showing relevant restriction endonuclease sites and positions of probes, using similar nomenclature as Fig. 1. B. 2DNAGE autoradiographs for fragments from the regions of mTTF binding sites bs1 and bs2, probed as indicated. Major pauses indicated by red arrows. C. The location of replication pauses in the various fragments tested, based on mobility in the first electrophoretic dimension (inversely proportional to the logarithm of total strand-length), and on unidirectional replication, as determined previously [16], directionality as shown. Numbers refer to nucleotide positions in the mitochondrial genome. The mid-point of each fragment corresponds with the apex of the Y-arc, indicated by dashed lines. Multiple digests, as shown, enable unambiguous mapping of the major replication pauses to the mTTF binding sites.
(PDF)

Figure S2 A. Q-RT-PCR analysis of mTTF transcript levels in S2 cells after 3 days of dsRNA treatment against mTTF. B. Q-RT-PCR analysis of S2 cell mitochondrial transcripts transcribed in the opposite direction to that of replication fork passage, in untreated cells. C. Analysis of mtDNA copy number by Southern hybridization, following 5 d of dsRNA treatment against mTTF or an inert dsRNA targeted against GFP, as shown. Biological replicate samples were digested with *XhoI*, run on a 0.35% agarose gel, and probed successively for mtDNA and nuclear rDNA using PCR-derived probes for nt 9363–9888 (ND4/ND4L region) of mtDNA (NCBI Accession U37541) and nt 1953–2446 of Drosophila rDNA (NCBI Accession M21017), labeled by random-primed synthesis in presence of α-^32^P-dCTP and hybridized under the standard conditions [26]. D. Indicated bands corresponding to nuclear rDNA and mtDNA fragments were quantitated by phosphorimaging, with background subtraction, and plotted as means + SD, normalized to the values for the control cells (i.e. those treated with dsRNA against GFP). E. Q-PCR analysis of mtDNA copy number after treatment with dsRNAs used in this study and the one used by Roberti et al [52].
(PDF)

Figure S3 Comparison of 2DNAGE patterns produced by NdeI digestion in control and mTTF knockdown cells. A. Top panels reproduced from Fig. 2F of this paper (cells knocked down for mTTF). Bottom panels reproduced from Fig. 6 of [26] (control cells), alongside cartoon diagrams of the gels. Red arrows indicate the broken replication intermediates produced by S1 nuclease digestion of material from control cells, but already present in material from mTTF knockdown cells. b – bubble arc (initiation arc), p1, p2 – major replication pauses 1 (at mTTF binding site bs1) and 2 (at mTTF binding site bs2), dY – double-Y arc, Y – y-arc, t – termination intermediates. For explanation of these arcs, see standard references on 2DNAGE [19]–[23], [60]. B. The same gels from part A, shown alongside the corresponding gels for mTerf5 knockdown cells, reproduced from Fig. 6B. To make the gels more easily comparable, the gel images from panel are slightly cropped for alignment, whilst those from Fig. 6B have been slightly stretched in the vertical dimension to compensate for slightly altered running conditions.
(PDF)

Figure S4 Verification of mtTTF knockdown *in vivo*, at the RNA and protein levels. A. Q-RT-PCR of mTTF mRNA in control (w^1118^ ; +/+ ; *da-GAL4*/+) and mTTF knockdown (w^1118^ ; *UAS-mTTF-RNAi*/+ ; *da-GAL4*/+) larvae. B. Western blot analysis of mTTF knockdown at the protein level *in vivo*. Protein extracts (25 µg) from males and females of different control strains and mTTF knockdown larvae (KD). Red arrow denotes the polypeptide corresponding with mTTF.
(PDF)

Figure S5 2DNAGE analysis of larval mtDNA from mTTF RNAi and control strains. Note the spreading of the signal along the Y-arc (red arrows) in larvae knocked down for mTTF, compared with the more specific pause in control larvae, plus the increase in X-structures (blue arrows).
(PDF)

Figure S6 RusA distinguishes the products of random collisions of the replication and transcription machineries from those of protein-mediated replication pausing. A. Unregulated collisions of the replication (blue) and transcription (green) machineries result in fork reversal, creating chicken-foot structures containing a Holliday (4-way) junction, that require a restart pathway to resume DNA replication. Protein-mediated replication pausing does not lead to fork reversal, and the paused Y-intermediate does not contain a Holliday junction. The Holliday junction formed upon fork reversal is susceptible to digestion by RusA. B. RusA cuts symmetrically in either of two modes (blue or orange arrows), degrading the chicken-foot species into 1n and sub-1n linear fragments, consistent with Fig. 4B for the case of cells knocked down for mTTF. Note that genuine Y-form intermediates are unaffected by RusA, and persist.
(PDF)

Figure S7 Comparison of RusA effect on replication intermediates in control and mTTF knockdown cells (3 days after start of dsRNA treatment). Equal amounts of material from single mtDNA preparations were cut with ClaI and then treated with 0, 25 and 100 nM concentrations of RusA (see Materials and Methods). Samples were hybridized together on the same membrane: differences in Y-arc intensity are therefore caused only by RusA. Equal loading and comparability of exposures are confirmed by the similar signal intensities of uncut linear partials (orange arrows). Two exposures of material from mTTF knockdown cells are shown for better visualization of 1n spot and Y-arc signal. The lower panels represent a similar exposure as for control material.
(PDF)

Figure S8 Additional material is resolved by 2DNAGE following RNase H treatment, as indicated. A. HindIII digest hybridized to probe 3 (two different exposures). B. ClaI digest hybridized to probe 6. Panels from Fig. 5 were run on the same gel and probed on the same membrane. Alongside each 2D gel panel is shown the ethidium bromide stained first-dimension gel prior to casting of the second dimension gel, confirming equal loading. Following RNaseH treatment, novel arcs appear against an essentially unaltered background of other species resolved on the gels.
(PDF)

Figure S9 Absence of RNAi cross-reaction between mTerf5 and mTTF. Q-RT-PCR analysis of mTerf5 and mTTF transcript in cells after 1–5 days of RNAi treatment against mTerf5. Knockdown of mTerf5 mRNA is effective within 24 h, whereas there is no knockdown of mTTF mRNA (if anything a possible slight increase, but certainly no classical off-target effect).
(PDF)

Figure S10 Comparable exposures of gels (ClaI fragments and probes as indicated) from control cells and mTerf5 knockdown cells, illustrating the increase in signal from the specific pause sites (red arrows), after mTerf5 knockdown. In particular, the relative strengths of the pause signals can be judged with reference to other features of the gels that are essentially invariant, such as the linear ‘partial’ species lying on the diagonal (orange arrows). These ‘partials’ are not the result of insufficient activity of the restriction enzyme, which is always present in excess, but are a constant feature of these gels and are seen in all digests. Note that the signals from these partials are actually slightly stronger in the control panels than in the corresponding gels from mTerf5 knockdown cells, in which the pauses are clearly stronger. The invariant part of the standard Y-arc may also be used for reference, for example the initial segment of the Y-arc, which is well separated from the pause region in the bs1-containing fragment (green arrow), or the apex of the Y-arc in the bs2-containing fragment (blue arrow).
(PDF)

Table S1 Oligonuclelotides.
(PDF)

Text S1 Supplemental materials and methods and reference.
(DOC)
