# Supplementary figures and images for: Mitochondrial Transcription Terminator Family Members mTTF and mTerf5 Have Opposing Roles in Coordination of mtDNA Synthesis
Source: PLoS Genet. 2013 Sep 19;9(9):e1003800. doi: 10.1371/journal.pgen.1003800 (PMC3778013; doi:10.1371/journal.pgen.1003800)

**A**

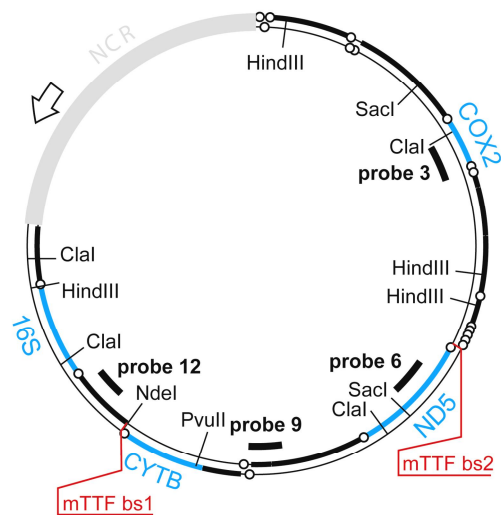

**B**

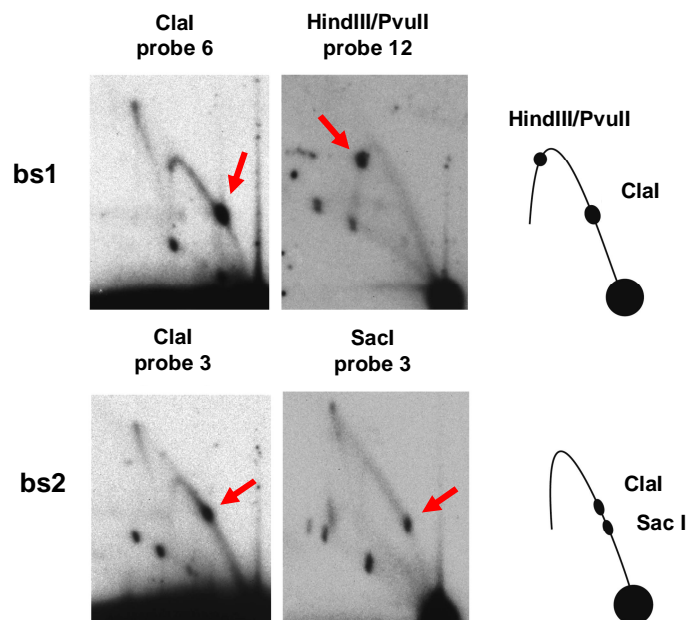

**C**

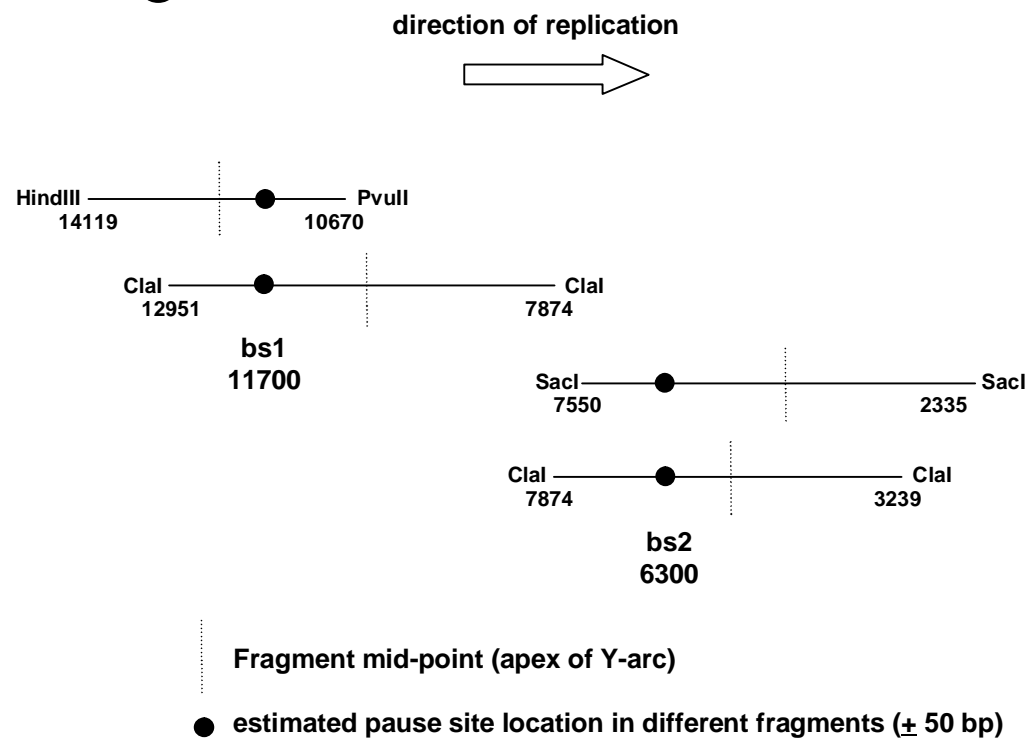

Figure S1, Jöers et al

Supplement: Figure S1 — Location of replication pause sites coincides with mTTF binding sites. A. Map of Drosophila mitochondrial genome showing relevant restriction endonuclease sites and positions of probes, using similar nomenclature as Fig. 1. B. 2DNAGE autoradiographs for fragments from the regions of mTTF binding sites bs1 and bs2, probed as indicated. Major pauses indicated by red arrows. C. The location of replication pauses in the various fragments tested, based on mobility in the first electrophoretic dimension (inversely proportional to the logarithm of total strand-length), and on unidirectional replication, as determined previously [16], directionality as shown. Numbers refer to nucleotide positions in the mitochondrial genome. The mid-point of each fragment corresponds with the apex of the Y-arc, indicated by dashed lines. Multiple digests, as shown, enable unambiguous mapping of the major replication pauses to the mTTF binding sites. (PDF) [file pgen.1003800.s001.pdf]

**A**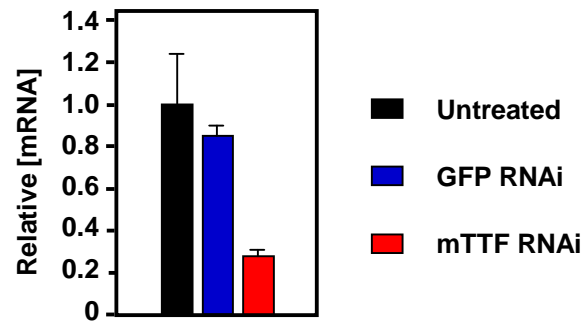**B**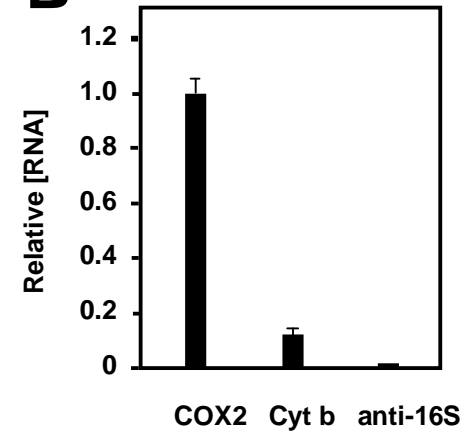**C**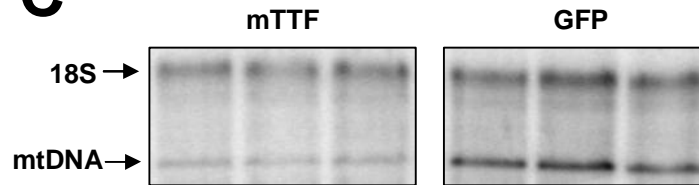**D**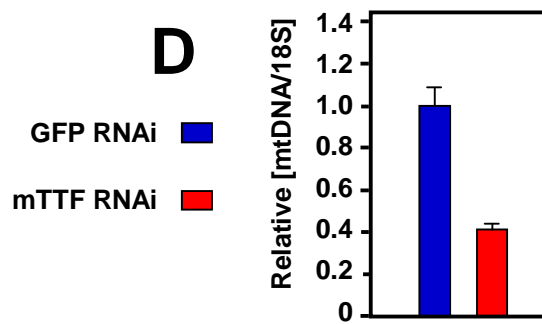**E**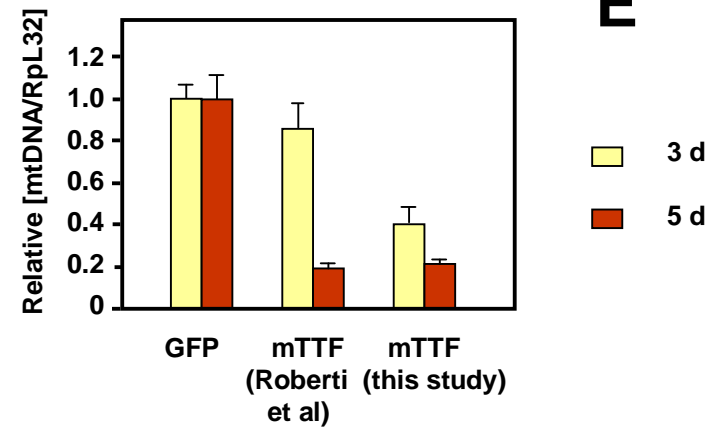

Supplement: Figure S2 — A. Q-RT-PCR analysis of mTTF transcript levels in S2 cells after 3 days of dsRNA treatment against mTTF. B. Q-RT-PCR analysis of S2 cell mitochondrial transcripts transcribed in the opposite direction to that of replication fork passage, in untreated cells. C. Analysis of mtDNA copy number by Southern hybridization, following 5 d of dsRNA treatment against mTTF or an inert dsRNA targeted against GFP, as shown. Biological replicate samples were digested with XhoI, run on a 0.35% agarose gel, and probed successively for mtDNA and nuclear rDNA using PCR-derived probes for nt 9363–9888 (ND4/ND4L region) of mtDNA (NCBI Accession U37541) and nt 1953–2446 of Drosophila rDNA (NCBI Accession M21017), labeled by random-primed synthesis in presence of α-32P-dCTP and hybridized under the standard conditions [26]. D. Indicated bands corresponding to nuclear rDNA and mtDNA fragments were quantitated by phosphorimaging, with background subtraction, and plotted as means + SD, normalized to the values for the control cells (i.e. those treated with dsRNA against GFP). E. Q-PCR analysis of mtDNA copy number after treatment with dsRNAs used in this study and the one used by Roberti et al [52]. (PDF) [file pgen.1003800.s002.pdf]

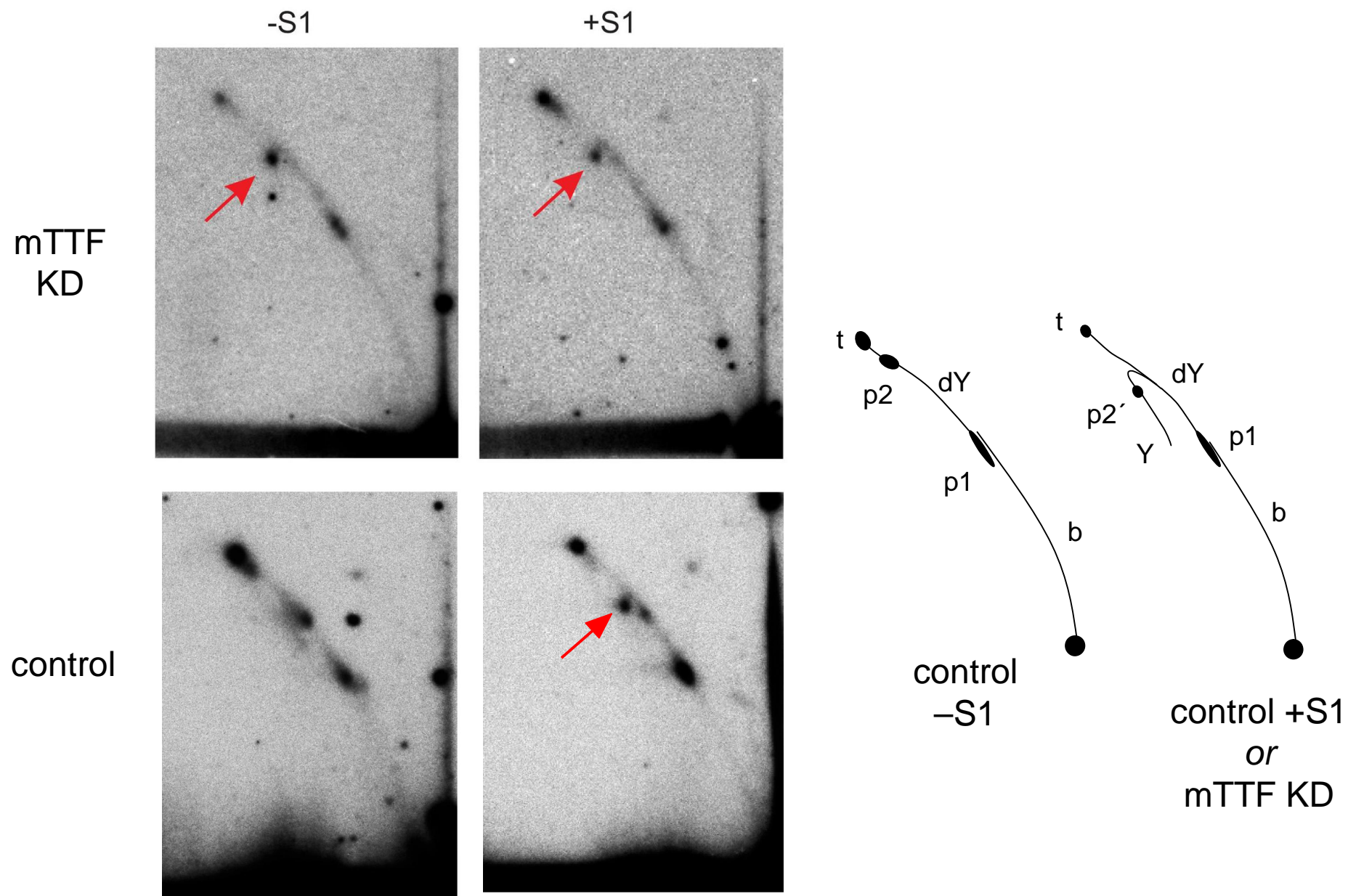

Figure S3, Jöers et al, page 1 of 2

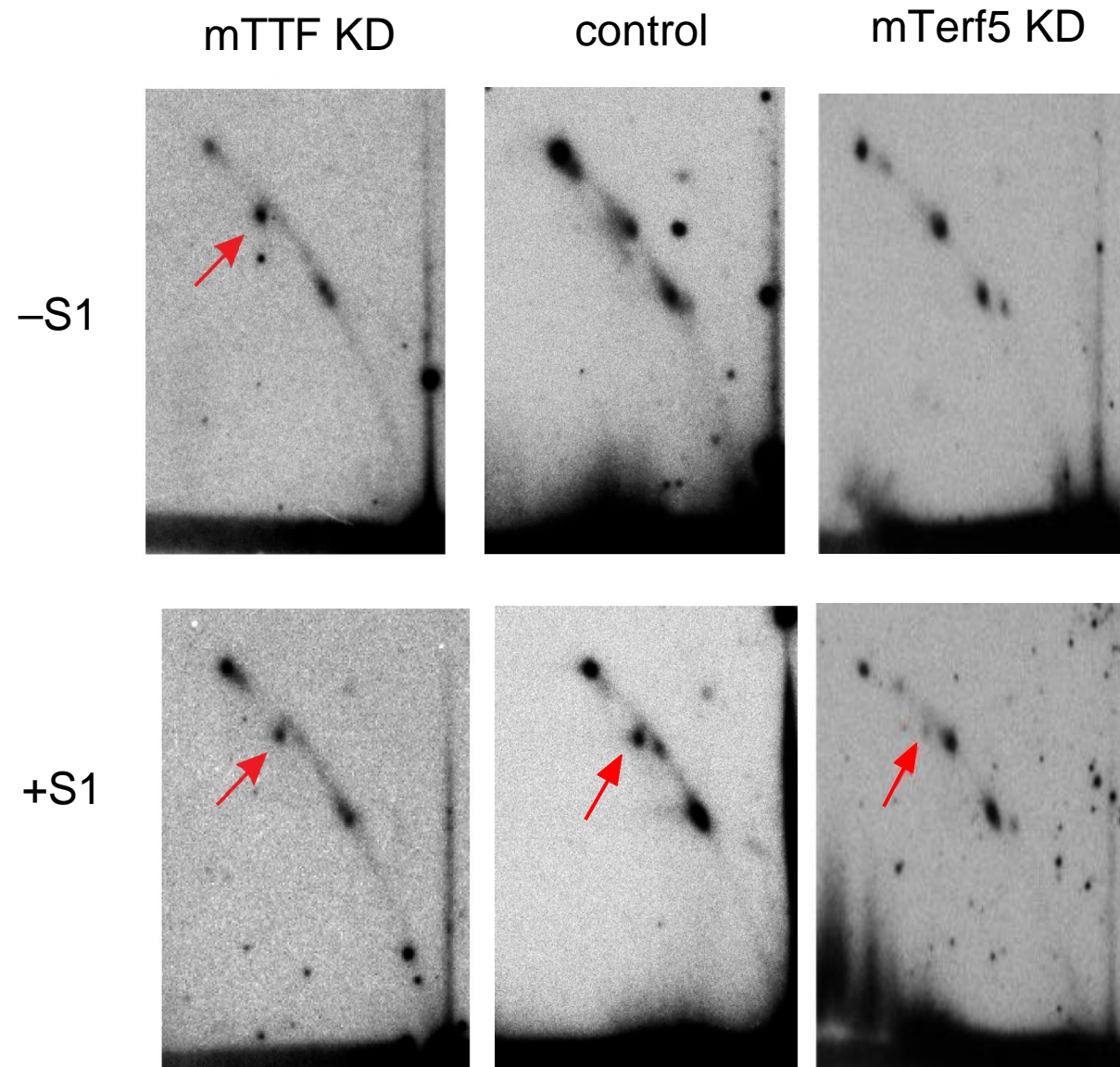

Figure S3, Jöers et al, page 2 of 2

Supplement: Figure S3 — Comparison of 2DNAGE patterns produced by NdeI digestion in control and mTTF knockdown cells. A. Top panels reproduced from Fig. 2F of this paper (cells knocked down for mTTF). Bottom panels reproduced from Fig. 6 of [26] (control cells), alongside cartoon diagrams of the gels. Red arrows indicate the broken replication intermediates produced by S1 nuclease digestion of material from control cells, but already present in material from mTTF knockdown cells. b – bubble arc (initiation arc), p1, p2 – major replication pauses 1 (at mTTF binding site bs1) and 2 (at mTTF binding site bs2), dY – double-Y arc, Y – y-arc, t – termination intermediates. For explanation of these arcs, see standard references on 2DNAGE [19]–[23], [60]. B. The same gels from part A, shown alongside the corresponding gels for mTerf5 knockdown cells, reproduced from Fig. 6B. To make the gels more easily comparable, the gel images from panel are slightly cropped for alignment, whilst those from Fig. 6B have been slightly stretched in the vertical dimension to compensate for slightly altered running conditions. (PDF) [file pgen.1003800.s003.pdf]

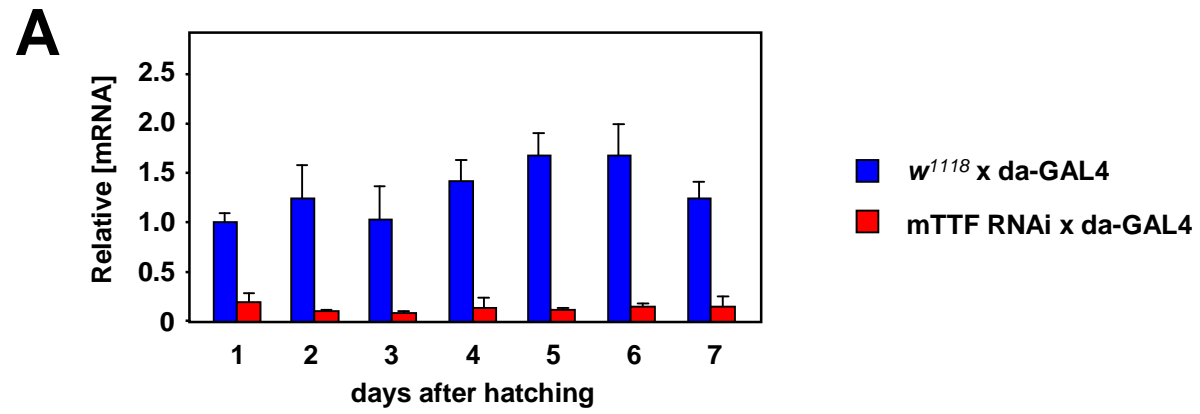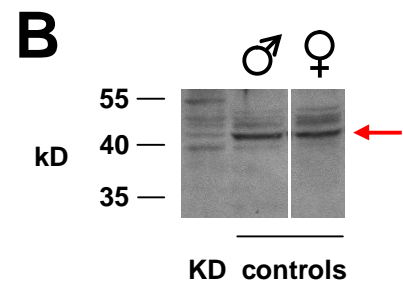

Figure S4, Jöers et al

Supplement: Figure S4 — Verification of mtTTF knockdown in vivo, at the RNA and protein levels. A. Q-RT-PCR of mTTF mRNA in control (w1118 ; +/+ ; da-GAL4/+) and mTTF knockdown (w1118 ; UAS-mTTF-RNAi/+ ; da-GAL4/+) larvae. B. Western blot analysis of mTTF knockdown at the protein level in vivo. Protein extracts (25 µg) from males and females of different control strains and mTTF knockdown larvae (KD). Red arrow denotes the polypeptide corresponding with mTTF. (PDF) [file pgen.1003800.s004.pdf]

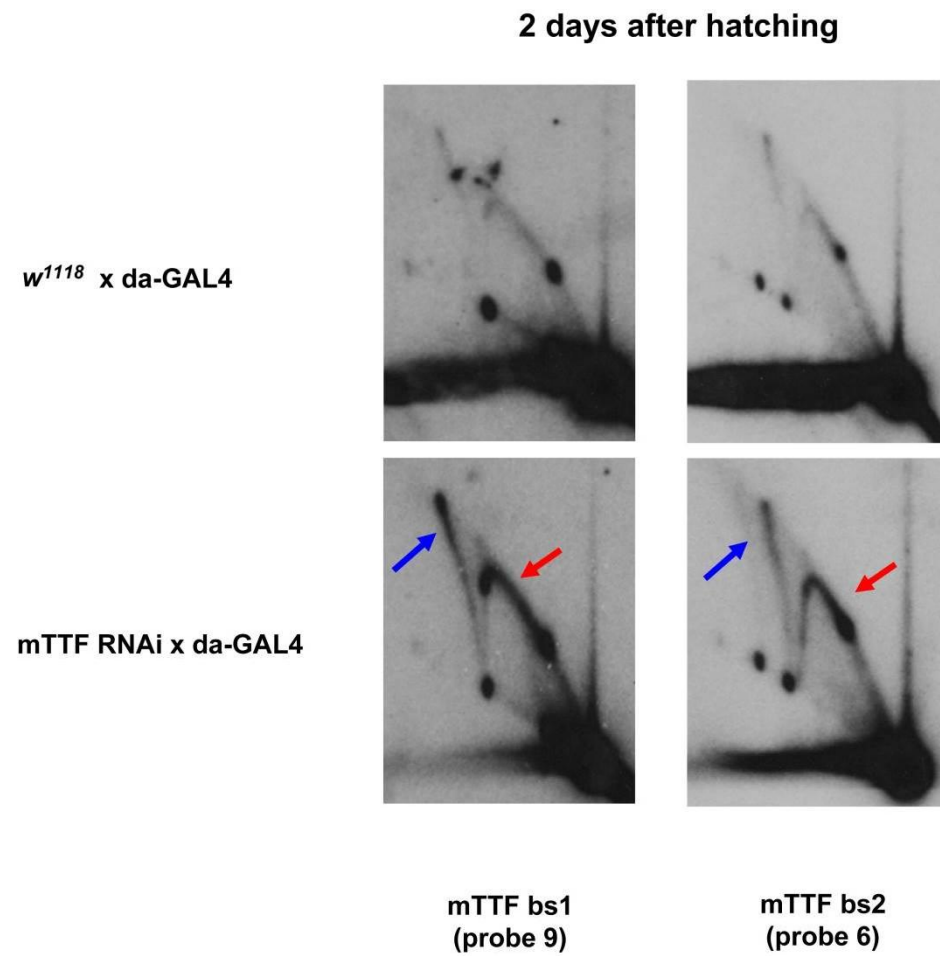

Figure S5, Jöers et al

Supplement: Figure S5 — 2DNAGE analysis of larval mtDNA from mTTF RNAi and control strains. Note the spreading of the signal along the Y-arc (red arrows) in larvae knocked down for mTTF, compared with the more specific pause in control larvae, plus the increase in X-structures (blue arrows). (PDF) [file pgen.1003800.s005.pdf]

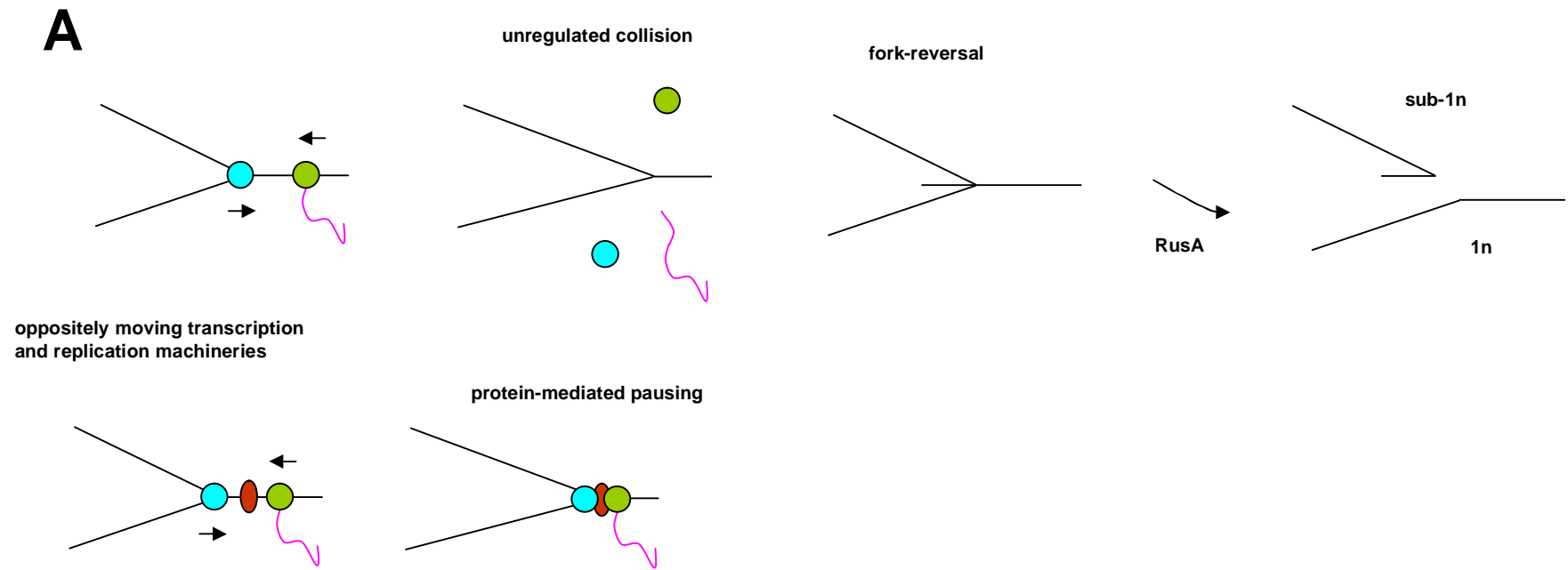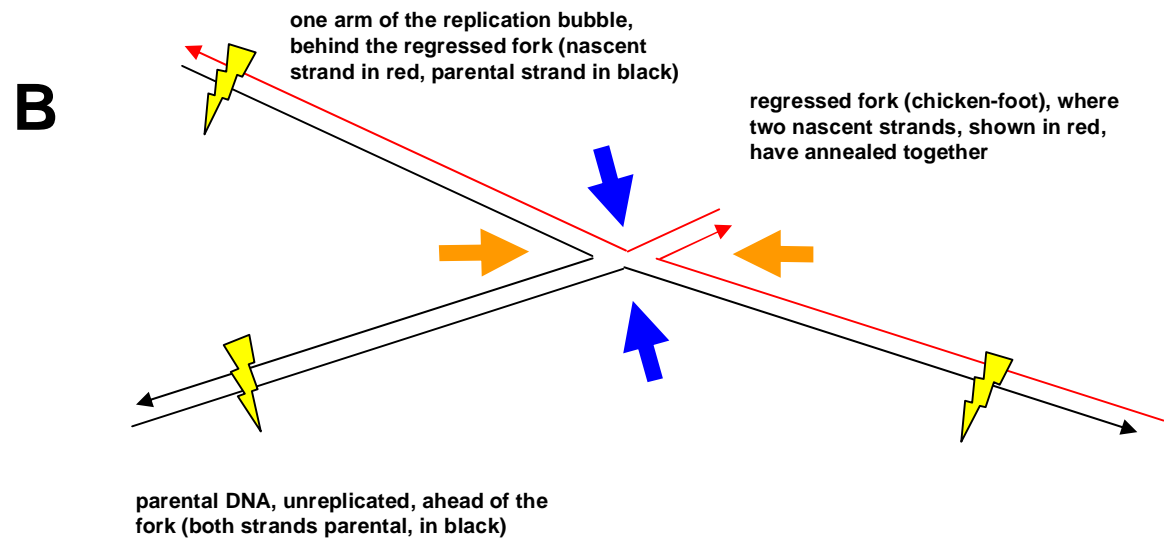

Figure S6, Joers et al

Supplement: Figure S6 — RusA distinguishes the products of random collisions of the replication and transcription machineries from those of protein-mediated replication pausing. A. Unregulated collisions of the replication (blue) and transcription (green) machineries result in fork reversal, creating chicken-foot structures containing a Holliday (4-way) junction, that require a restart pathway to resume DNA replication. Protein-mediated replication pausing does not lead to fork reversal, and the paused Y-intermediate does not contain a Holliday junction. The Holliday junction formed upon fork reversal is susceptible to digestion by RusA. B. RusA cuts symmetrically in either of two modes (blue or orange arrows), degrading the chicken-foot species into 1n and sub-1n linear fragments, consistent with Fig. 4B for the case of cells knocked down for mTTF. Note that genuine Y-form intermediates are unaffected by RusA, and persist. (PDF) [file pgen.1003800.s006.pdf]

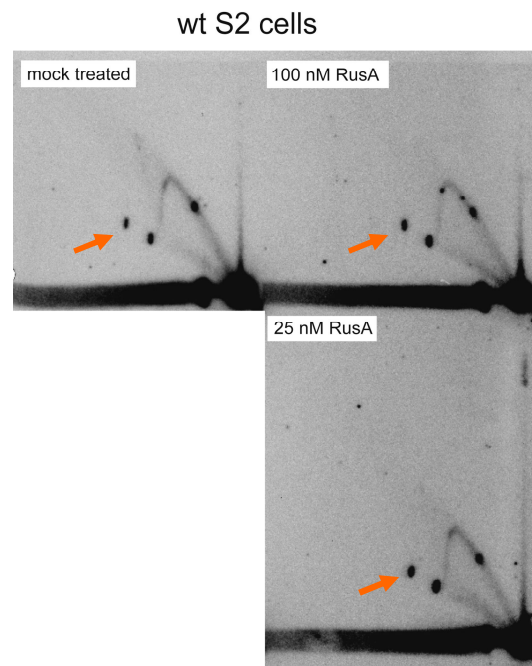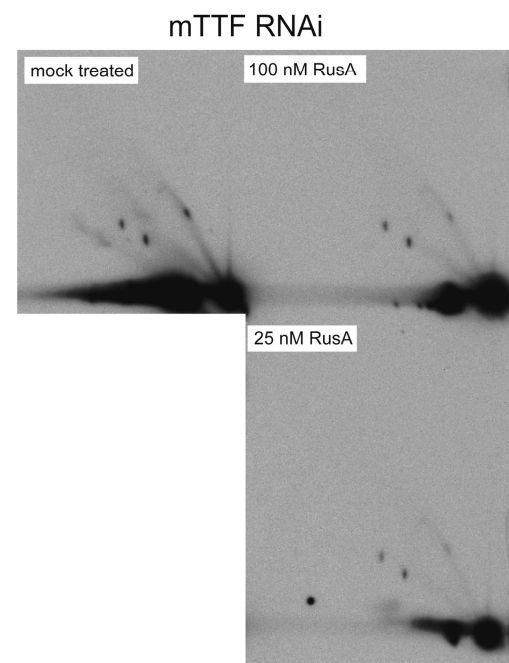

short expo

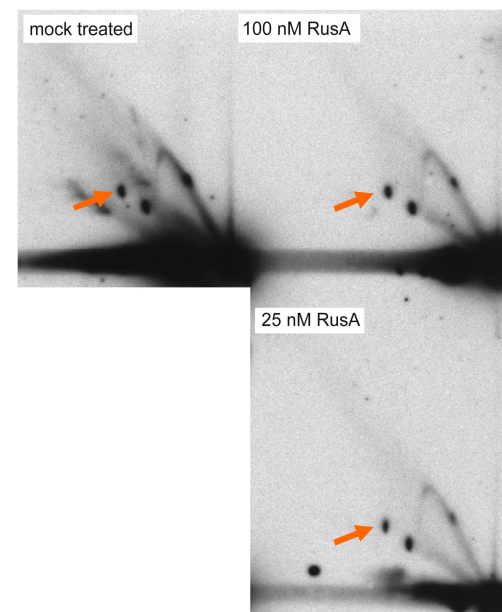

long expo

Figure S7, Jöers et al

Supplement: Figure S7 — Comparison of RusA effect on replication intermediates in control and mTTF knockdown cells (3 days after start of dsRNA treatment). Equal amounts of material from single mtDNA preparations were cut with ClaI and then treated with 0, 25 and 100 nM concentrations of RusA (see Materials and Methods). Samples were hybridized together on the same membrane: differences in Y-arc intensity are therefore caused only by RusA. Equal loading and comparability of exposures are confirmed by the similar signal intensities of uncut linear partials (orange arrows). Two exposures of material from mTTF knockdown cells are shown for better visualization of 1n spot and Y-arc signal. The lower panels represent a similar exposure as for control material. (PDF) [file pgen.1003800.s007.pdf]

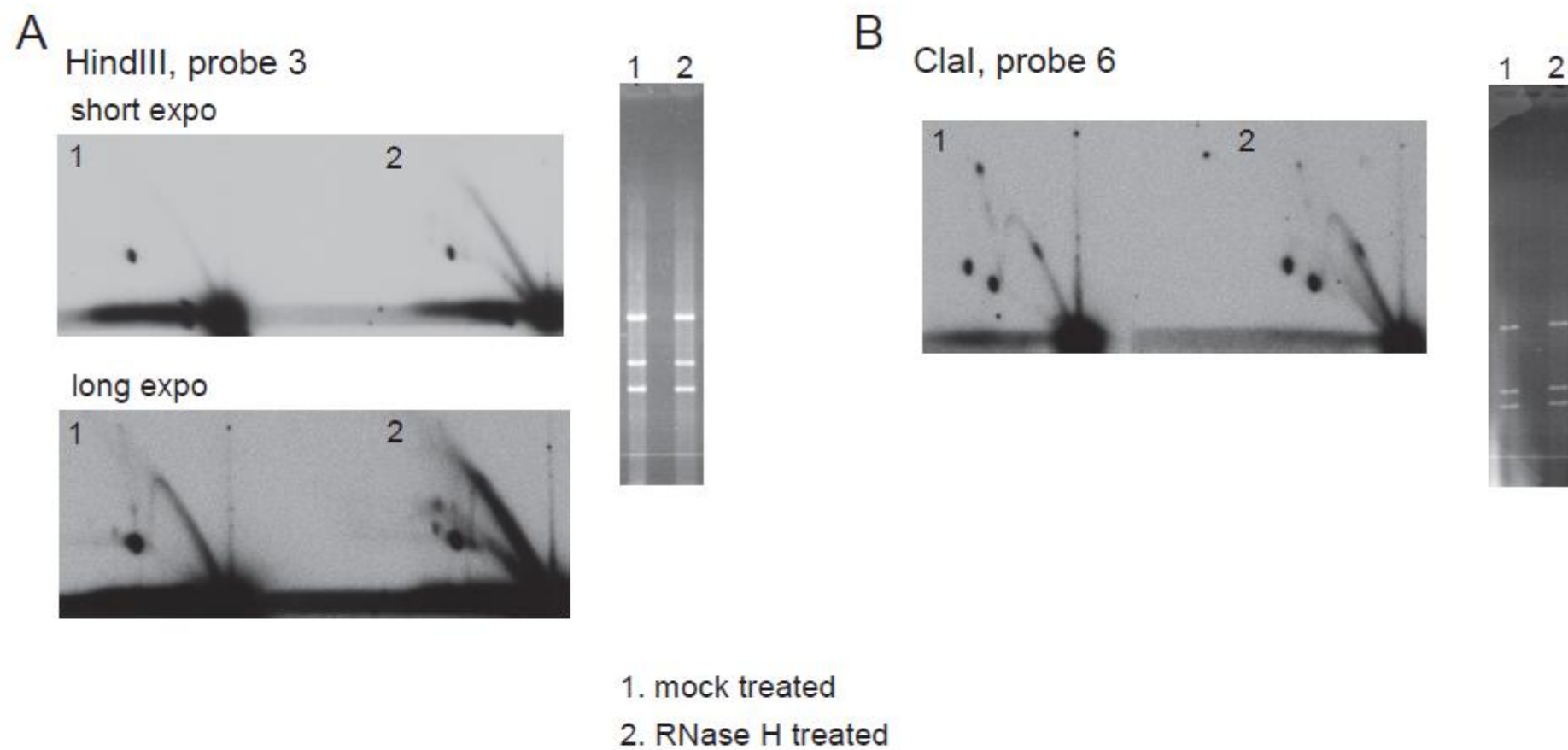

Supplemental Figure S8, Jôers et al

Supplement: Figure S8 — Additional material is resolved by 2DNAGE following RNase H treatment, as indicated. A. HindIII digest hybridized to probe 3 (two different exposures). B. ClaI digest hybridized to probe 6. Panels from Fig. 5 were run on the same gel and probed on the same membrane. Alongside each 2D gel panel is shown the ethidium bromide stained first-dimension gel prior to casting of the second dimension gel, confirming equal loading. Following RNaseH treatment, novel arcs appear against an essentially unaltered background of other species resolved on the gels. (PDF) [file pgen.1003800.s008.pdf]

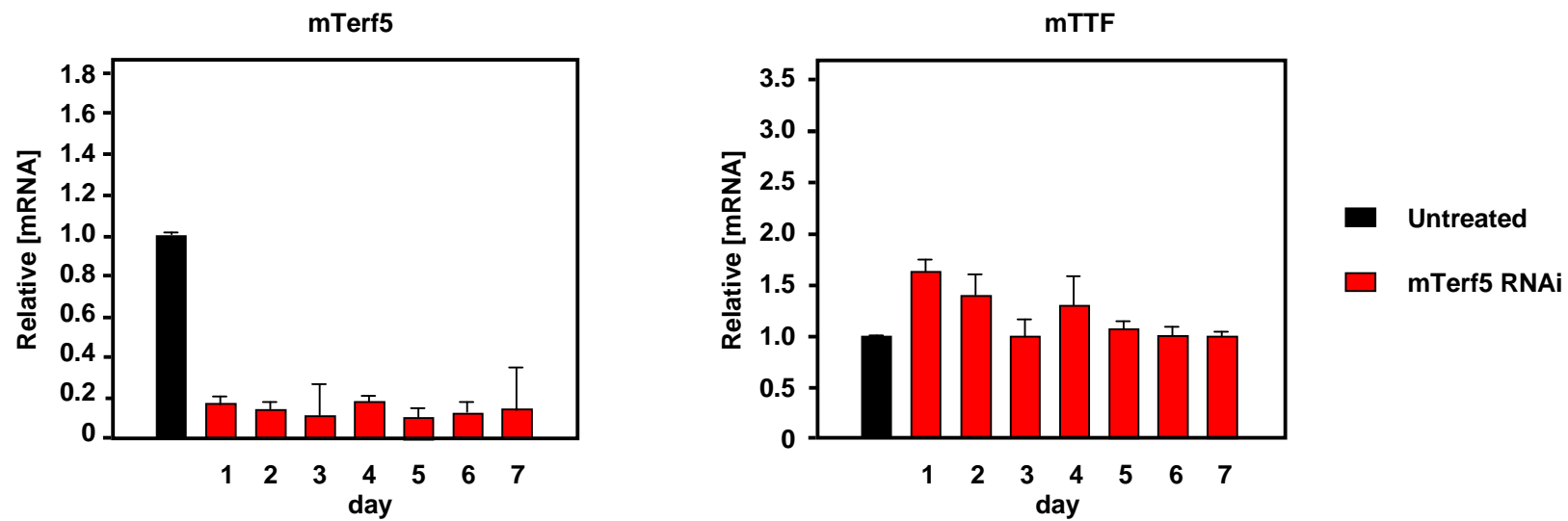

Supplement: Figure S9 — Absence of RNAi cross-reaction between mTerf5 and mTTF. Q-RT-PCR analysis of mTerf5 and mTTF transcript in cells after 1–5 days of RNAi treatment against mTerf5. Knockdown of mTerf5 mRNA is effective within 24 h, whereas there is no knockdown of mTTF mRNA (if anything a possible slight increase, but certainly no classical off-target effect). (PDF) [file pgen.1003800.s009.pdf]

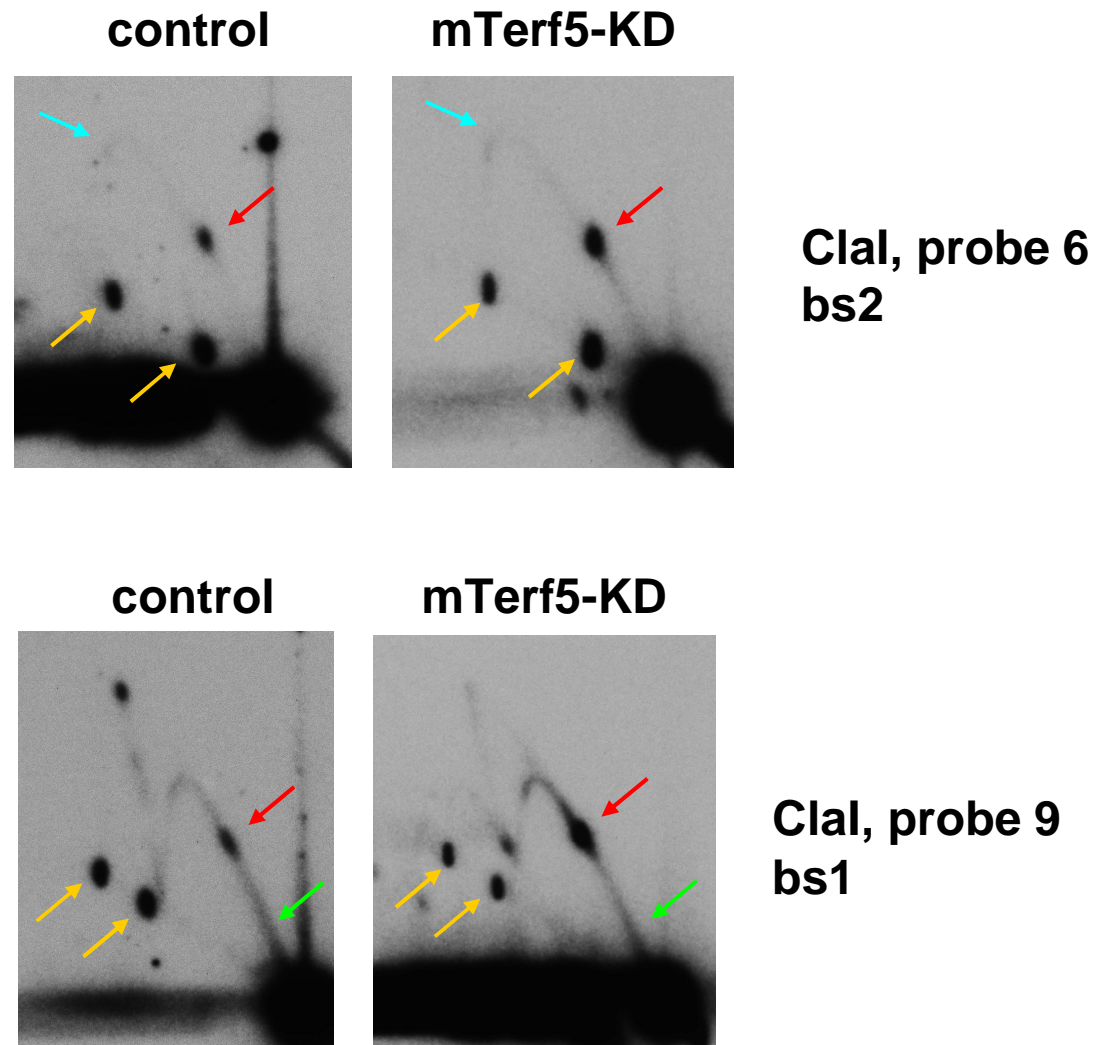

Figure S10, Jöers et al

Supplement: Figure S10 — Comparable exposures of gels (ClaI fragments and probes as indicated) from control cells and mTerf5 knockdown cells, illustrating the increase in signal from the specific pause sites (red arrows), after mTerf5 knockdown. In particular, the relative strengths of the pause signals can be judged with reference to other features of the gels that are essentially invariant, such as the linear ‘partial’ species lying on the diagonal (orange arrows). These ‘partials’ are not the result of insufficient activity of the restriction enzyme, which is always present in excess, but are a constant feature of these gels and are seen in all digests. Note that the signals from these partials are actually slightly stronger in the control panels than in the corresponding gels from mTerf5 knockdown cells, in which the pauses are clearly stronger. The invariant part of the standard Y-arc may also be used for reference, for example the initial segment of the Y-arc, which is well separated from the pause region in the bs1-containing fragment (green arrow), or the apex of the Y-arc in the bs2-containing fragment (blue arrow). (PDF) [file pgen.1003800.s010.pdf]
