# Supplementary material for: Mitochondrial Transcription Terminator Family Members mTTF and mTerf5 Have Opposing Roles in Coordination of mtDNA Synthesis
Source: PLoS Genet. 2013 Sep 19;9(9):e1003800. doi: 10.1371/journal.pgen.1003800 (PMC3778013; doi:10.1371/journal.pgen.1003800)
Supplement: Table S1 — Oligonuclelotides. (PDF) [file pgen.1003800.s011.pdf]

Supplemental Table S1: oligonucleotide primers

Target gene	Primer name	Sequence (5´ to 3´)	Purpose
CG5924 (twinkle)	5-1M-dTwinkle	ATGAGACGCGCCGGTTTAATCAAACCGCTG	1st round PCR for dsRNA
	3-222T-dTwinkle	TTTTGTTCATCGCACCGTGAATCAGCAGCC	1st round PCR for dsRNA
	5-T7-34E-dTwinkle:	GAATTAATACGACTCACTATAGGGAGAAGGAGTGCTCACTAGACCCCAAG	2nd round PCR for dsRNA
	3-T7-201R-dTwinkle	GAATTAATACGACTCACTATAGGGAGAGTCGATCGCCCAGATATAACACC	2nd round PCR for dsRNA
tamas (Polg)	5-T7-tamas	GAATTAATACGACTCACTATAGGGAGAGGCGGGTCAAGCCTC	PCR for dsRNA
	3-T7- tamas	GAATTAATACGACTCACTATAGGGAGACCCTCGCTGACGCAC	PCR for dsRNA
CG7175	5-cg7175	GGAGCCTTGGACAGCTGGCG	1st round PCR for dsRNA
	3-cg7175	CTTCCTTAGCCGCTCGGCGG	1st round PCR for dsRNA
	5-T7-cg7175	GAATTAATACGACTCACTATAGGGAGAAGAGCCGCTGAATGCCAGCT	2nd round PCR for dsRNA
	3-T7-cg7175	GAATTAATACGACTCACTATAGGGAGAGCGTGTAAACGCGCCACAGC	2nd round PCR for dsRNA
GFP	5-T7-eGFP	GAATTAATACGACTCACTATAGGGAGAACGTAAACGGCCACAAGTTCAGC	PCR for dsRNA
	3-T7-eGFP	GAATTAATACGACTCACTATAGGGAGAGGGTGTTCTGCTGGTAGTGGTCG	PCR for dsRNA
Tfam	5-1M-dTfam	ATGATCTACACCACAACACTGATGTCCTCG	1st round PCR for dsRNA
	3-stop-dTfam	CTATATATCTTTGGAGGCCAGCGTCTTGCG	1st round PCR for dsRNA
	5-T7-74P-dTfam	GAATTAATACGACTCACTATAGGGAGACGCCGCGACCAAAGAAACCGCTG	2nd round PCR for dsRNA
	3-T7-240L-dTfam	GAATTAATACGACTCACTATAGGGAGAAGCTCCATCTCCTTGCGCGACTC	2nd round PCR for dsRNA

mTTF	5-dmmTTF-RA	ATGATTAGAAGCCTTCTGCGCAGCTTTGAG	1st round PCR for dsRNA
	3-dmmTTF-RA	GGCGACACATTGTAGTCGATCATGTGCTGC	1st round PCR for dsRNA
	5-T7-dmmTTF	GAATTAATACGACTCACTATAGGGAGACCCAATGCACTGCAGCAGGCGCC	2nd round PCR for dsRNA
	3-T7-dmmTTFnew	GAATTAATACGACTCACTATAGGGAGACGCTGTGGCATCGCATCCCTTTC	2nd round PCR for dsRNA
	3-mTTF-RA2	TCCTTCTGATACACTTTGCGGATTTCTTTC	3´ primer for 1st round PCR for dsRNA (Roberti et al)
	5-T7-mTTFpc	GAATTAATACGACTCACTATAGGGAGAGAAAACTGGTGGGTGCTC	2nd round PCR for dsRNA (Roberti et al)
	3-T7-mTTFpc	GAATTAATACGACTCACTATAGGGAGAGATACACTTTGCGGATTTC	2nd round PCR for dsRNA (Roberti et al)
	5-DmTTF short2	ACACCGTGCGAGTTACCAAGATCA	Q-RT-PCR
	3-DmTTF short2	GCGCTCCTTTGTGGTTTTCAGGC	Q-RT-PCR
RpL32	RpL32F	TGTGCACCAGGAACTTCTTGAA	Q-RT-PCR，mtDNA copy number analysis by Q-PCR
	RpL32A	AGGCCCAAGATCGTGAAGAA	Q-RT-PCR，mtDNA copy number analysis by Q-PCR

16S	lrRNA-for	ACCTGGCTTACACCGGTTTG	Strand-specific Q-RT-PCR, RT (and PCR) primer for antisense-strand transcript quantitation, , mtDNA copy number analysis by Q-PCR
	lrRNA-rev	GGGTGTAGCCGTTCAAATTT	Strand-specific Q-RT-PCR, RT (and PCR) primer for sense-strand transcript quantitation, mtDNA copy number analysis by Q-PCR
ND5	5-ND5	GGGTGAGATGGTTTAGGACTTG	Strand-specific Q-RT-PCR, RT (and PCR) primer for antisense-strand transcript quantitation
	3-ND5	AAGCTACATCCCCAATTCGAT	Strand-specific Q-RT-PCR, RT (and PCR) primer for sense-strand transcript quantitation

Cyt b	5-cytb	GAAAATTCCGAGGGATTCAA	Strand-specific Q-RT-PCR, RT (and PCR) primer for antisense-strand transcript quantitation
	3-cytb	AACTGGTCGAGCTCCAATTC	Strand-specific Q-RT-PCR, RT (and PCR) primer for sense-strand transcript quantitation
COX2	5-COII	AAAGTTGACGGTACACCTGGA	Strand-specific Q-RT-PCR, RT (and PCR) primer for antisense-strand transcript quantitation
	3-COII	TGATTAGCCCCACAGATTTC	Strand-specific Q-RT-PCR, RT (and PCR) primer for sense-strand transcript quantitation
Probe	Dm27F	AATGAATTGCCTGATAAAAAGGATTAC	Probe for hybridization
	Dm394R	ATTAAATTATTATTATCTCTTAATAGGG	Probe for hybridization

Probe 3	Dm3276F	AACTATTTTACCAGCAATTATTTTACT	Probe for hybridization
	Dm3840R	CAGTCATCTAATGAAGAGTTATTTCTA	Probe for hybridization
Probe 6	Dm6801F	AAATCAATCAATTTAATATTCTACCTC	Probe for hybridization
	Dm7378R	ATTAACAATATTTATAGCTGGATTAGG	Probe for hybridization
Probe 9	Dm9363F	AATCCATAAGATAATATATCACAACCT	Probe for hybridization
	Dm9888R	ATAATCTTATTTTTGATTTACAAGACC	Probe for hybridization
Probe 12	Dm12075F	GCTAATGAAATAGATACTCAAACTAAA	Probe for hybridization
	Dm12584R	TTTATTAGAACGAAAAGTTTTAGGATA	Probe for hybridization
