# Supplementary material for: Mitochondrial Transcription Terminator Family Members mTTF and mTerf5 Have Opposing Roles in Coordination of mtDNA Synthesis
Source: PLoS Genet. 2013 Sep 19;9(9):e1003800. doi: 10.1371/journal.pgen.1003800 (PMC3778013; doi:10.1371/journal.pgen.1003800)
Supplement: Text S1 — Supplemental materials and methods and reference. (DOC) [file pgen.1003800.s012.doc]

**SUPPLEMENTAL TEXT S1**

**SUPPLEMENTAL MATERIALS AND METHODS**

**Flies, cell-lines and culture**

Schneider’s S2 cell-line [63] was cultured in Schneider’s Medium (Sigma-Aldrich), including 10% fetal bovine serum, at 25 °C, usually with the addition of 100 U/ml penicillin/streptomycin (Lonza). Cells were passaged every 3-4 d at a density of 0.5 x 10^6^ cells/ml. Standard *Drosophila* strains *w^1118^* and da-GAL4, plus the mTTF RNAi line 101656 from the Vienna Drosophila RNAi Center (VDRC), were cultured as described previously [87]. For larval wet weight measurements larvae were collected from food plates attached to mating chambers for no more than three hours containing approximately 200 – 250 flies. Food plates were saturated with water and individual larvae were collected with forceps from the slurry, followed by brief drying on a pad of paper towels. Weight was calculated as a mean from three individual 1.5 ml Eppendorf tubes containing larvae. The number of larvae collected per tube varied from 30 to 60 depending on the developmental stage.

**dsRNA constructs and transfection**

cDNA was synthesized from S2 cell RNA using the High Capacity cDNA Reverse Transcription kit (Applied Biosystems), under manufacturer’s recommended conditions and was then amplified in two steps to create gene-specific dsRNAs, as follows. In the first step, 700 bp amplicons were generated by PCR with *Taq* DNA polymerase (Fermentas), using primer pairs as detailed in Table S1. Amplification conditions for first round PCR were: 95 °C for 3 min, then 30 cycles of 95 °C for 30 s, Tm-5 °C for 30 s and 72 °C for 1 min. PCR products were purified by NucleoSpin Extract II spin-columns (MACHEREY-NAGEL), their concentration determined by NanoDrop 2000 (Thermo Scientific), then used as template in a second, nested reaction using chimeric primers, in which gene-specific segments defining ~500 bp amplicons were preceded by the T7 promoter sequence (see Table S1). Amplification conditions for second round PCR were: 95 °C for 3 min, then 5 cycles of 95 °C for 10 s, 55 °C for 30 s and 72 °C for 1 min and a further 25 cycles of 95 °C for 10 s, 60 °C for 30 s and 72 °C for 1 min. To create control dsRNAs against GFP and tamas, PCR used appropriate cDNA plasmids, as template. After product purification, double-stranded RNA (dsRNA) was synthesized from each template using the MEGAscript T7 Kit (Ambion) according to the manufacturer’s instructions. dsRNAs were precipitated with lithium chloride according to manufacturer’s instructions, resuspended in nuclease-free water and stored at –20 °C. 0.5 x 10^6^ S2 cells were transfected with 4 μg of each dsRNA added to 0.5 ml of culture medium, and grown for the times indicated in figures and legends. Where transfections were to be continued for >3 d, cells were passaged every 3 d and fresh dsRNA was added.

**DNA and RNA extraction**

For mtDNA copy-number analysis, total DNA was isolated from confluent S2 cells grown in 24-well plates (0.5 ml culture medium per well), as follows. Cells were harvested by centrifugation at 1,200 *g_max_* for 7 min, washed once in 1 ml of PBS at room temperature, repelleted and resuspended in 125 μl of DNA extraction buffer (75 mM NaCl, 25 mM EDTA, pH 8.0), to which were added 12.5 μl of 10 % SDS and 2.5 μl of 20 μg/μl proteinase K (Fermentas). After mixing and overnight incubation at 37 °C, DNA was recovered by two rounds of vigorous extraction with 2 vol of 25:24:1 phenol:chloroform:isoamyl alcohol (Amresco), followed by centrifugation at 5,000 *g_max_* for 15 min and final precipitation with 0.2 vol of 10 M ammonium acetate and 2 vol of ethanol at –20 °C for 10 min. DNA was pelleted at 16,000 *g_max_* for 15 min, washed once with 70% ethanol, air-dried and resuspended in 50 μl TE buffer. For mtDNA copy-number analysis from adult flies or larvae, total DNA was extracted by homogenizing 30-50 animals, depending on developmental stage, in 400 μl of buffer A (100 mM Tris-HCl, 100 mM EDTA, 100 mM NaCl, 0.5% SDS, pH 7.5) After incubation at 65 °C for 30 min, 800 μl of buffer B (1.43 M potassium acetate, 4.29 M LiCl) was added and the suspension incubated for 1 h, after mixing on ice. Debris was pelleted by centrifugation at 12,000 *g_max_* for 15 min at room temperature, and the supernatant transferred to a fresh tube. Nucleic acids were precipitated with 600 μl of isopropanol and pelleted by centrifugation at 12,000 *g_max_* for 15 min at room temperature, followed by washing with 70% ethanol. For 2DNAGE, mitochondrial DNA (nucleic acid) was extracted from flies collected at different developmental stages according to the previously published procedure [26]. For DNA extraction from S2 cells for 2DNAGE, confluent cells were harvested from between one and three 175 cm2 flasks, washed once with medium, then with ice-cold PBS, with recovery in each case by centrifugation for 3 min at 600 *g_max_*, then resuspended in 2 ml of 0.1 x cell homogenization buffer (CHB, 40 mM Tris-HCl, 25 mM NaCl, 50 mM MgCl_2_, pH 7.8) and incubated on ice for 6 min (at 4 °C, as for all subsequent steps in the procedure). The cell suspension was lysed in a Dounce homogenizer by 10-15 strokes of a tight-fitting pestle, after which 200 μl of 10 x CHB was added. The homogenate was centrifuged for 3 min at 1,200 *g_max_*. The supernatant was transferred to a fresh tube and centrifugation repeated. Crude mitochondria were pelleted in a fresh tube at 14,000 *g_max_* for 3 min, resuspended in 500 μl of HB, then processed by sucrose-density gradient centrifugation and nucleic acid extraction as for the material from flies. DNA was also prepared from crude mitochondria (i.e. before sucrose gradient), for mtDNA topology analysis by Southern blotting (Fig. 2D). RNA was extracted from >5 x 10^6^ S2 cells grown in 6-well plates, using the TRIzol reagent (Invitrogen). Cells were harvested by centrifugation at 1,200 gmax for 7 min at and resuspended in 1 ml of TRIzol reagent at room temperature by pipetting, then stored at –80 °C prior to further processing. After thawing at room temperature for 5 min, 0.2 ml of chloroform was added, samples shaken by hand, incubated for 2-3 min, then centrifuged at 12000 *g_max_* for 15 min at 4 °C. RNA was recovered from the aqueous phase by adding 0.5 ml isopropanol for 10 min at room temperature, followed by centrifugation at 12000 *g_max_* for 10 min at 4°C. After washing with 75% ethanol, air-dried pellets were resuspended in 50 μl RNase-free water. RNA concentrations were measured by NanoDrop 2000 (Thermo Scientific). RNA was extracted from adult flies or larvae (between 30 and 50 individuals depending on developmental stage) collected into a 1.5 ml Eppendorf tube and ground with a Teflon-coated pestle in 1 ml of TRI Reagent (Molecular Research Center). The homogenate was incubated at room temperature for 5 min before adding 200 μl of chloroform, and then for a further 3 min. Debris was removed by centrifugation at 12,000 gmax for 15 min at 4 °C and 600 μl of the supernatant was precipitated with 500 μl of isopropanol in a fresh tube. After 10 min of incubation at room temperature nucleic acid was pelleted at 12,000 *g_ma_*_x_ for 10 min at 4 °C, and washed with 70% ethanol. This was followed by treatment with RNase-free DNase I (Fermentas) according to the manufacturer’s protocol and subsequent re-precipitation of RNA overnight at –20 °C using 1 vol of 3 M sodium acetate, 2.5 volumes of ethanol, followed by centrifugation at 11,000 *g_max_* for 15 min at 4°C, and washing with 70% ethanol.

**Fluorescence microscopy of mtDNA nucleoids**

S2 cells were seeded into 24-well plates (0.5 ml, 10^6^ cells/ml) and allowed to attach for 30 min at 25 °C, after which the medium was replaced with medium containing 4 μg of dsRNA against a given target gene, or an inert dsRNA against GFP as a control. After culture for 3 d at 25 °C, cells were passaged into 24-well plates (0.5 ml, 10^6^ cells/ml), 4 μg of dsRNA targeted against Tfam were added, and cells cultured for a further 2 d at 25 °C. In test experiments, spiking the dsRNA in this way produced no significant (additional) drop in mtDNA copy number during the time of the experiment, but did enable mtDNA nucleoids to be much more clearly visualized in control cells, presumably because of the documented effects of the Tfam expression level on mtDNA topology ([90], see below). Note also that spiking against Tfam was not used in any experiments in which mtDNA copy number, replication intermediates or RNA levels were analyzed by other methods (Q-PCR, Southern blotting, 2DNAGE). Nucleoids were visualized after cells were split 1:4 into 6-well plates, and allowed to attach for 30 min at 25 °C. The medium was then replaced with 1 ml normal medium containing 7.5 μl of Quant-iT™ PicoGreen (Invitrogen) and cells were incubated for 30 min at 25 °C. After two rinses with normal medium, cells were incubated for a further 2 h in 1 ml of normal medium, then rinsed once more with 2 ml of medium, to remove debris, prior to imaging by fluorescence microscopy (Olympus BX61W1 with cell^M 2.1 software).

**Q-RT-PCR measurement of RNA levels**

Q-RT-PCR was performed essentially as described previously [36]. RNA was reverse-transcribed using the High Capacity cDNA Reverse Transcription kit (Applied Biosystems) under manufacturer’s recommended conditions. Each reaction was carried out in a final volume of 20 μl using 2 μg of total RNA and either Random Primers provided in the kit or 20 pmol of strand-specific mtDNA primers (see Table S1). Real-Time PCR was performed using the SYBR Green PCR MasterMix (Applied Biosystems) and the ABI StepOnePlus™ Real-Time PCR System (Applied Biosystems), in ∆∆Ct configuration. Cytosolic ribosomal protein RpL32 was used as endogenous control (see Table S1). Each reaction was run in triplicate and contained 2 μl of a 1:200 dilution of the reverse transcription reaction along with 1 μl (10 pmol) of gene specific primers in a final reaction volume of 20 μl. Amplification conditions were: 95 °C for 20 s, then 50 cycles of 95 °C for 30 s and 60 °C for 30 s. To ensure only a single product was amplified, melting curve analysis was performed by StepOne Software version 2.1 (Applied Biosystems). The relative amounts of target transcripts were normalized to RpL32. For each amplicon, the mean ratio value was obtained from three real-time RT–PCR assays, using RNA obtained from independent biological replicates.

**mtDNA copy number analysis by Q-PCR**

Relative quantitation of mtDNA content was performed as for Q-RT-PCR, except that 2 μl of total DNA (4 μg/ml) was used as template, plus primers for mitochondrial 16S rDNA (Table S1). Normalization was again to the nuclear gene RpL32.

**1D and 2D neutral agarose gel electrophoresis and Southern blot-hybridization**

Standard one-dimensional electrophoresis used 0.35% or 0.6% agarose gels run in TBE buffer at 1.8 V/cm for 20 h, with 1 μg per lane of nucleic acid isolated from crude mitochondria. Two-dimensional electrophoresis also used TBE buffer in both dimensions, with typically 5-10 μg of total nucleic acid per track. Restriction endonuclease (Fermentas except for *Cla*I, from Promega) reactions were incubated at 37 °C for 4 h with 4 units of enzyme per μg of mitochondrial nucleic acid, in manufacturer’s recommended buffers. Subsequent treatment with RNase H (Fermentas, 0.5 u per μg of nucleic acid, 1h at 37 °C) or S1 (0,2 u per μg of nucleic acid, 2 min at room temperature) were also carried out according to the enzyme manufacturer’s protocol, after stopping the initial restriction digest and removing enzyme by phenol/chloroform extraction and ethanol precipitation. 5 μg of nucleic acids were treated with 25 nM RusA (a kind gift from Peter McGlynn) in reaction buffer (25 mM Tris-HCl, 1 mM DTT, 2.1 mM BSA, 10% glycerol, 10 mM MgCl_2_, pH 8.0) at 37 °C for 30 min. Control samples (mock treated) were treated with identical conditions but without RNase H, S1 or RusA. mtDNA cut by restriction enzymes with a single digestion site in the genome was analyzed by 2DNAGE according to the previously published protocol [16]. Smaller mtDNA fragments were separated in the first dimension without ethidium bromide in 0.4% agarose, 1.8 V/cm for 20 h at 4°C. After the first dimension the gel was stained with ethidium bromide (300 ng/ml in TBE) and documented. Individual sample lanes were cut and placed into the second-dimension tray, rotated through 90º. The second-dimension gel containing 300 ng/ml ethidium bromide was cast around it at 55 °C, and electrophoresis initiated with constant buffer circulation in 1.0% agarose, 10 V/cm for 6 h at 4°C. Blotting, probe synthesis, hybridization, washing and autoradiography were carried out as described previously [16], except where indicated in legends.

**Protein extraction and Western blotting**

Total protein extracts were prepared from *Drosophila* adults or larvae, and analyzed by SDS-PAGE and Western blotting as described previously [87], using a customized rabbit antiserum raised against peptides from mTTF (21^st^ Century Biologicals),

**SUPPLEMENTAL REFERENCE**

90. Pohjoismäki JL, Wanrooij S, Hyvärinen AK, Goffart S, Holt IJ, et al. (2006) Alterations to the expression level of mitochondrial transcription factor A, TFAM, modify the mode of mitochondrial DNA replication in cultured human cells. Nucleic Acids Res 34: 5815-5825.
